# Digital Technologies for Women’s Pelvic Floor Muscle Training to Manage Urinary Incontinence Across Their Life Course: Scoping Review

**DOI:** 10.2196/44929

**Published:** 2023-07-05

**Authors:** Stephanie J Woodley, Brittany Moller, Alys R Clark, Melanie D Bussey, Bahram Sangelaji, Meredith Perry, Jennifer Kruger

**Affiliations:** 1 Department of Anatomy School of Biomedical Sciences University of Otago Dunedin New Zealand; 2 Auckland Bioengineering Institute University of Auckland Auckland New Zealand; 3 School of Physical Education, Sport and Exercise Sciences University of Otago Dunedin New Zealand; 4 Southern Queensland Rural Health Brisbane Australia; 5 School of Physiotherapy University of Otago Wellington New Zealand

**Keywords:** apps, culture, life course, mobile health, mHealth, pelvic floor muscle training, urinary incontinence, women’s health, mobile phone

## Abstract

**Background:**

Women with urinary incontinence (UI) may consider using digital technologies (DTs) to guide pelvic floor muscle training (PFMT) to help manage their symptoms. DTs that deliver PFMT programs are readily available, yet uncertainty exists regarding whether they are scientifically valid, appropriate, and culturally relevant and meet the needs of women at specific life stages.

**Objective:**

This scoping review aims to provide a narrative synthesis of DTs used for PFMT to manage UI in women across their life course.

**Methods:**

This scoping review was conducted in accordance with the Joanna Briggs Institute methodological framework. A systematic search of 7 electronic databases was conducted, and primary quantitative and qualitative research and gray literature publications were considered. Studies were eligible if they focused on women with or without UI who had engaged with DTs for PFMT, reported on outcomes related to the use of PFMT DTs for managing UI, or explored users’ experiences of DTs for PFMT. The identified studies were screened for eligibility. Data on the evidence base for and features of PFMT DTs using the Consensus on Exercise Reporting Template for PFMT, PFMT DT outcomes (eg, UI symptoms, quality of life, adherence, and satisfaction), life stage and culture, and the experiences of women and health care providers (facilitators and barriers) were extracted and synthesized by ≥2 independent reviewers.

**Results:**

In total, 89 papers were included (n=45, 51% primary and n=44, 49% supplementary) involving studies from 14 countries. A total of 28 types of DTs were used in 41 primary studies, including mobile apps with or without a portable vaginal biofeedback or accelerometer-based device, a smartphone messaging system, internet-based programs, and videoconferencing. Approximately half (22/41, 54%) of the studies provided evidence for or testing of the DTs, and a similar proportion of PFMT programs were drawn from or adapted from a known evidence base. Although PFMT parameters and program compliance varied, most studies that reported on UI symptoms showed improved outcomes, and women were generally satisfied with this treatment approach. With respect to life stage, pregnancy and the postpartum period were the most common focus, with more evidence needed for women of various age ranges (eg, adolescent and older women), including their cultural context, which is a factor that is rarely considered. Women’s perceptions and experiences are often considered in the development of DTs, with qualitative data highlighting factors that are usually both facilitators and barriers.

**Conclusions:**

DTs are a growing mechanism for delivering PFMT, as evidenced by the recent increase in publications. This review highlighted the heterogeneity in types of DTs, PFMT protocols, the lack of cultural adaptations of most of the DTs reviewed, and a paucity in the consideration of the changing needs of women across their life course.

## Introduction

### Background

Pelvic floor muscle (PFM) dysfunction, which most commonly manifests as urinary incontinence (UI), pelvic organ prolapse, and pain, is a major and often unreported problem for women. UI, defined as “any involuntary leakage of urine” [1], affects between 25% and 45% of women worldwide, yet the true prevalence is likely to be higher, with women underreporting UI because of the associated shame and embarrassment [1]. UI substantially affects quality of life (QoL) in relation to both women’s physical and mental health and well-being and also represents a major economic burden (eg, costs associated with routine care and treatment) [2].

PFM training (PFMT), which includes exercises to increase PFM strength and endurance, is recommended as the first choice for managing UI, especially stress UI [1]. PFMT can be undertaken by women to maintain pelvic health by preventing the onset of UI or can cure or improve symptoms and enhance QoL in adult and older women, including during pregnancy and the postpartum period [3,4]. However, despite its effectiveness, approaches to PFMT vary across communities and countries, and maintaining exercise programs, which are often undertaken at home, is difficult [5]. In addition, many women avoid seeking treatment for UI based on the belief that UI is an inevitable consequence of aging or childbirth, or the perception that little can be done to improve symptoms or QoL, or because of limited access to health services [6].

Digital technologies (DTs; such as the World Wide Web; eHealth; and mobile health [mHealth], including SMS text messaging and apps) provide an avenue for women with UI to seek guidance with PFMT and potentially improve their symptoms and QoL [5-8]. To elicit health benefits, women require access to DTs based on the best scientific evidence. However, although the market appears to be flooded with PFMT apps, few have been scientifically validated in terms of content, quality, or appropriateness [9,10]. In addition, knowing whether PFMT delivered via DTs is sound from a clinical perspective is equally important, but there is a lack of information as to whether PFMT in this context is based on contemporary evidence [11]. This is potentially compounded by the notion that few mHealth apps have been developed in collaboration with key stakeholders, such as women experiencing UI or health care professionals [12].

Factors such as age and culture may influence how women engage with PFMT DTs. For example, there is evidence that women are more vulnerable to developing UI at certain stages in life, including (1) young athletic women, particularly those participating in high-impact sports [13]; (2) during and after pregnancy, when one-third of women giving birth for the first time have UI, which may persist for at least 3 months post partum; (3) menopause, where a peak in UI occurs; and (4) older women (UI prevalence ranges from 43% to 77%), particularly those in residential care, where UI is a substantial risk factor for falls [1]. On the basis of this evidence, age-appropriate and specific PFMT programs seem imperative to best cater to women, yet there appears to be a distinct lack of information related to the uptake of DTs to manage UI at different stages in life [11]. Culture, which encompasses particular spiritual, intellectual, and emotional features, including lifestyle, value systems, traditions, and beliefs [14], not only affects how women interact with DTs [15,16] but also shapes their experiences and attitudes toward UI [15-17]. Although it is essential to understand how culture may affect the use of and engagement with PFMT DTs, with reference to UI, it is unclear whether the experiences and needs of women from different cultures or ethnic groups are considered when developing these types of DTs.

### Objectives

Several systematic reviews have recently been published in this field, mostly focusing on the effectiveness of PFMT DTs in terms of improving symptoms of UI and QoL along with adherence to the prescribed PFMT program [7,8,18-21]. In this context, knowledge of the quality and content of PFMT DTs is also important, as is an understanding of whether such DTs are designed for women across their life course and take into account the cultural contexts and experiences of women and other relevant stakeholders. The main aim of this scoping review was to provide a narrative synthesis of digital health technologies used for women’s PFMT to manage UI. The key objectives of this review were to (1) explore whether PFMT DTs follow best-practice guidelines and describe outcomes related to their use, (2) establish whether DTs have been designed for PFMT at specific stages in life or consider culture, and (3) describe users’ experiences of DTs for PFMT.

## Methods

This scoping review was conducted in accordance with the Joanna Briggs Institute methodological framework [22] and the PRISMA-ScR (Preferred Reporting Items for Systematic Reviews and Meta-Analyses extension for Scoping Reviews) [23]. A protocol was prospectively registered with the Open Science Framework [24].

### Search Strategy

Following an initial search in PubMed, a systematic search of 7 electronic databases (AMED, CINAHL, Embase, MEDLINE, SPORTDiscus, Scopus, and PsycINFO) was conducted to identify relevant literature (from inception to December 2021). Key search concepts related to (1) DTs (eg, smartphones, cell phones, apps, telemedicine, and mHealth), (2) UI, (3) PFMT or exercise, and (4) key life stages (eg, pregnancy and menopause). Hand searching of reference lists of included articles, as well as citation tracking (eg, Web of Science for the last 5 years), was used to identify additional articles that may have been eligible for screening and inclusion (Multimedia Appendix 1).

### Study Selection Criteria

#### Population

Studies were included if they (1) focused on women aged ≥14 years with or without UI who were using or had used some form of DT to engage in PFMT, (2) evaluated outcomes related to the use of PFMT DTs for managing UI, or (3) explored users’ experiences of DTs for PFMT. Studies were excluded if the research focus was on women with overactive bladder or enuresis or neurological conditions, they reported collective data for men and women unless the data specific to women could be extracted separately, they used biofeedback devices that were not connected to an app or any other form of eHealth, and they were published in a language other than English for which a translation could not be acquired (eg, through Google Translate).

#### Concept

This review considered studies that explored PFMT delivered via DTs for the management of UI—with a focus on the evidence base for DTs and PFMT—the life course, culture, and users’ experiences*.* The use of DTs for health (PFMT) is defined as “a broad umbrella term encompassing eHealth (which includes mHealth), as well as emerging areas, such as the use of advanced computing sciences in ‘big data,’ genomics, and artificial intelligence” [25]. In addition to eHealth, other applications such as wearable devices with a digital component (eg, a vaginal biofeedback probe connected to a mobile app), telehealth, and personalized medicine are encompassed within the scope of DTs.

#### Context

Studies that met the previously defined criteria were included to establish the widest coverage of information related to PFMT delivered via DTs for managing UI. This encompassed a large and heterogeneous group of women with or without UI, health care providers (HCPs) or researchers, and other disciplines (eg, IT experts). Any type of health care setting (eg, primary care and community) or discipline (eg, physiotherapy and general practitioners) was considered.

#### Study Design

Research involving quantitative and qualitative study designs and other forms of gray publications, such as opinion pieces, editorials, conference abstracts, theses, and case studies or series, were considered [26].

### Study Selection

Titles and abstracts were independently screened by 5 authors (AC, BS, JK, MB, and SW) using web-based software (Covidence systematic review software; Veritas Health Innovation). The full texts were read and assessed by 2 of the 3 authors (BM, BS, and SW), with discrepancies resolved through consensus or discussion with another member of the team.

### Data Extraction and Verification

A customized template was developed in Microsoft Word (Microsoft Corp) and piloted on 5 of the included studies. Data were independently extracted into the template by 5 authors (MB, AC, JK, BM, BS, and SW), transferred to a Microsoft Excel (Microsoft Corp) spreadsheet, and cross-checked by 2 authors (BS and SW). Any disagreements were resolved through consensus or consultation with a third reviewer when necessary.

The data extracted related to general study and participant characteristics included authors; year of publication; country; study aims; sample size; inclusion and exclusion criteria (intervention and comparator groups if relevant); age, gender, and level of education of participants; type of UI; and duration of symptoms.

To address the key objectives of this review, the data outlined in Textbox 1 were extracted.

**Data extracted from the included studies.**

- Data related to the digital technologies (DTs)
  Evidence base for or validation through previous testing
  Type and features of DTs—capacity to extract, educational features, gamification, reminders and reinforcements, social media and self-monitoring, and technical support [7,27]
- Data specific to pelvic floor muscle training (PFMT)
  Evidence base for the PFMT program
  Descriptions of PFMT, which were charted according to the 16 key elements in the PFMT variation of the Consensus on Exercise Reporting Template (CERT-PFMT) [28]. In the context of this review, there was overlap between some CERT-PFMT items and DT features. Item 1 (exercise equipment) in the CERT-PFMT refers to the DT of interest, which incorporates descriptions of the device and related features (eg, biofeedback and mobility requirements) [7]. Item 5 (adherence) is covered by “self-monitoring,” item 6 (motivation) relates to “reminders and reinforcements,” and item 10 (nonexercise components) equates to “educational features.” In the case of overlap, the data were extracted and synthesized under the umbrella of DT.
- Outcomes related to the use of PFMT DTs for managing urinary incontinence (UI), including UI symptoms, quality of life, and adherence to and satisfaction with the program
- Information related to key life stages and the culture of the women engaging with the PFMT DTs
- Experiences (facilitators and barriers) of women and health care providers with PFMT DTs for managing UI

### Data Synthesis

The included studies that shared common author teams or apps were grouped accordingly. Descriptive statistics were used to summarize the data (BM, BS, and SW). With the exception of the study protocols, the methodological quality of the included studies was independently appraised using the relevant Joanna Briggs Institute critical appraisal tools [29] by pairs of reviewers, with a third reviewer consulted to reach a consensus if required.

For qualitative studies or the qualitative components of mixed methods studies, thematic synthesis, with the development of analytical themes driven by our review questions (ie, deductive analysis), was used for data synthesis [30] (MP and SW). The analysis occurred over 3 steps, with the last step designed to present clear implications for HCPs and policy makers. First, coding of text segments from the results and discussion specific to the review objectives was performed from sections of the included articles. Next, the raw codes were grouped and named in an iterative manner to form descriptive themes (grouped by the study’s reported main themes and women’s or clinician’s perceptions of facilitators of and barriers to the use of DTs). Finally, analytical themes were generated from descriptive themes, and these analytical themes extended the synthesis beyond the conclusions of the included articles. Data were grouped for both barriers and facilitators under the headings of interactions between users and eHealth, interactions between users and PFMT exercises, and interactions between PFMT exercises and eHealth [31,32]. Although other tangential themes were generated, we presented the themes that were most coherently related to the study objectives.

### Deviations From the Protocol

Owing to the large number of papers retrieved, a decision was made to exclude systematic reviews, meta-analyses, and scoping reviews from the analysis, which represents a deviation from the study protocol. Similarly, because of the number of DTs included in this review, we did not classify the types of DTs using the World Health Organization (WHO) classification [33] or rate the apps using the Mobile App Rating Scale [34].

## Results

### Search Results and Characteristics of the Included Studies

**Figure 1:**
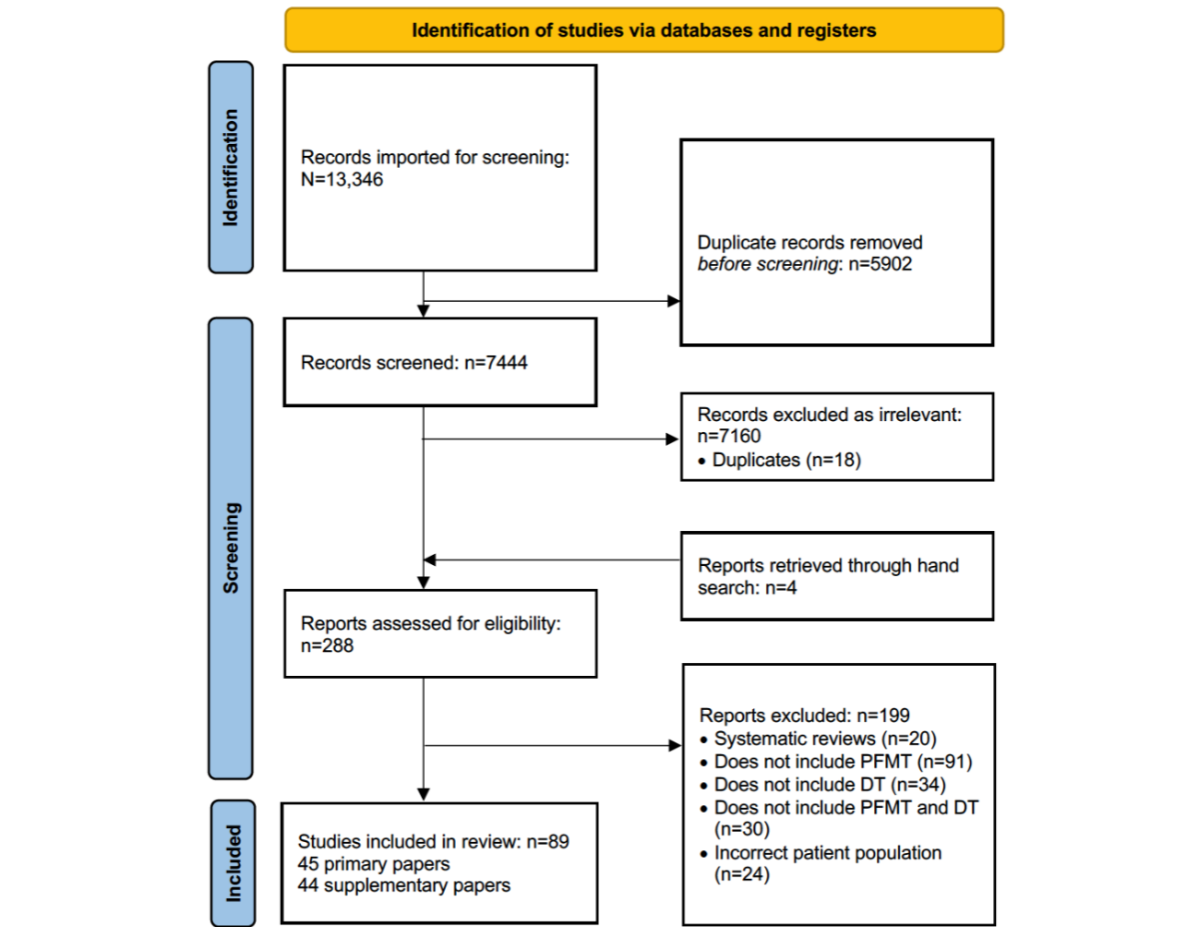
PRISMA (Preferred Reporting Items for Systematic Reviews and Meta-Analyses) study flow diagram—search for papers related to digital technologies (DTs) and pelvic floor muscle training (PFMT) for women.

**Figure 2:**
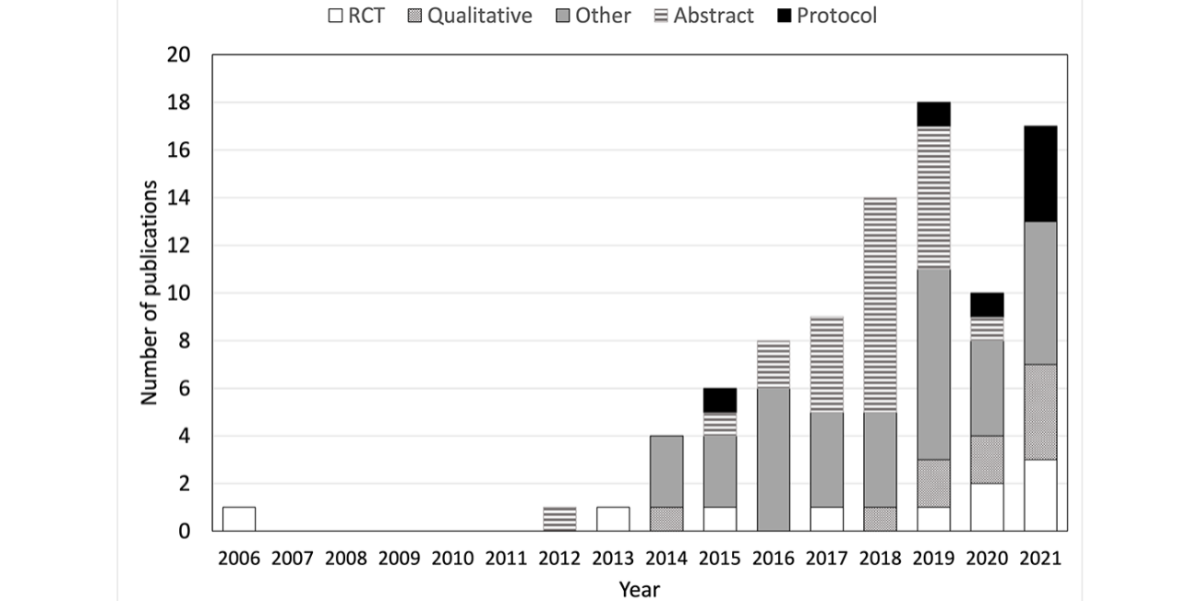
Publications included in this review presented by year of publication. RCT: randomized controlled trial.

From the 7444 records screened for titles and abstracts, and after the removal of duplicates, 288 (3.87%) full-text reports were reviewed (Figure 1). A total of 89 papers met the inclusion criteria, of which 45 (51%) were classified as primary papers, with the other 44 (49%) considered supplementary papers (Table S1 in Multimedia Appendix 2 [5,6,11,12,31,35-118]; Table S2 in Multimedia Appendix 2 presents the inclusion and exclusion criteria for the included studies [5,6,11,12,31,35-118]). Of the 45 primary studies, many were randomized controlled trials (RCTs; n=13, 29%), with various other designs including cross-sectional studies (n=7, 16%); qualitative studies (n=6, 13%); mixed methods studies combining either RCTs or quasi-experimental trials with qualitative research (n=4, 9%); quasi-experimental studies (n=4, 9%); cohort studies (n=4, 9%); case series (n=4, 9%); and a case report, case-control, and validation study; of these 45 studies, 6 (13%) were study protocols and 2 (4%) were published in a language other than English (Dutch [35] and Portuguese [36]). The supplementary articles consisted of follow-up studies, secondary analyses, associated abstracts reporting a subset of data from the primary article, and author comments and letters to the editor (eg, [89,90,92-99,102-106,108-118]). Publications in this area have increased rapidly since the 2010s, with most protocols registered since 2019 (Figure 2). The methodological quality was rated for 84% (38/45) of the primary studies and was predominantly high (15/38, 39%) or fair (14/38, 37%), with 24% (9/38) considered poor (Multimedia Appendix 3 [5,6,36-41,43-46,48-51,53,57-59,61-77,80]). Key methodological areas for consideration included blinding of participants, therapists, and outcome assessors; measuring outcomes in a valid and reliable way and identifying confounding factors (cross-sectional studies); and consecutive recruitment of participants (case series), although aspects such as double-blinding are recognized as problematic in pragmatic and clinical trials.

The studies originated in 14 countries, primarily from Europe (9/45, 20% from Sweden; 7/45, 16% from the Netherlands; 3/45, 7% from Spain; and 1/45, 2% from Germany), as well as the United States (6/45, 13%), the United Kingdom (4/45, 9%), Brazil (4/45, 9%), China (4/45, 9%, including Hong Kong), Australasia (4/45, 9%), Canada (1/45, 2%), Japan (1/45, 2%), and Malaysia (1/45, 2%). A total of 47% (21/45) of the studies had a total sample size of <50, 9% (4/45) recruited between 50 and 100 participants, and 44% (20/45) of the studies sampled >100 participants (2/20, 10% of which analyzed data from a sample of >10,000 women; Multimedia Appendix 3).

### Participant Characteristics

#### Age

The age of the participants ranged from 18 to 98 years, with approximately 35% of studies (16/45, 36%) including women in their 40s or 50s as the average age. The level of education of the recruited participants was stated for just over half (24/45, 53%) of the primary studies. Among these, most women in each study were shown to be educated at the university level.

#### UI in the Included Studies

Studies mostly recruited women with stress UI only (17/45, 38%) [5,6,37-51], whereas 7% (3/45) of the studies included those with stress and mixed UI if stress symptoms were predominant [52-54], and 24% (11/45) included a mixture of stress, urge, and mixed UI types [55-65]. In total, 11% (5/45) of the studies included healthy (continent) and incontinent participants [66-70], and 11% (5/45) did not clearly specify the UI type [71-75], and this was irrelevant in 7% (3/45) [36,70,80]. Only 2% (1/45) of the studies [76], a case study, looked at urge UI only. A total of 20% (9/45) of the studies documented the duration of women’s UI symptoms before their inclusion in the study. Of these, Asklund et al [5] required participants to have had symptoms for at least 6 months as part of their inclusion criteria. The remaining 18% (8/45) of the studies [38,40,43,46,51,61,64,65] showed variable durations, ranging from <3 months to 26 years (Multimedia Appendix 3).

#### Stage in Life

A total of 17 (38%) of the 45 studies reported life stage parameters: 29% (5/17) recruited women in the postpartum period [67-69,75,77]; 18% (3/17) recruited pregnant women [47,51,60]; 12% (2/17) included both pregnant and postpartum women [52,66]; 6% (1/17) included postmenopausal women [59]; and 24% (4/17) reported including a mixture of premenopausal, perimenopausal, postmenopausal, lactating, and postpartum participants [45,46,58,62]. A case study [76] included women reported as parous and Campbell et al [42] recruited athletic women for their RCT.

#### Cultural Context

Some studies developed the DTs for use by women in their specific countries (eg, Sweden [5], Japan [68], and Germany [70]), and the Tät has been translated into a number of different languages [56,63,78].

One group conducted a systematic review to explore variables that may influence adherence to PFMT DTs, which led to the development of the iPelvis app [11,57]. The authors emphasized the importance of considering ethnicity as part of a woman’s individuality, and as such, the avatar character within the iPelvis app can be ethnically matched to the woman by altering features such as skin color, the flag of the country, and cultural costumes, as well as age and stage in life (eg, pregnant or older adult). This concept was supported by Han et al [72], who stated that the information in apps needs to be formatted in a culturally relevant way to ensure that it is effective. The importance of ongoing research to evaluate apps in different and diverse cultural contexts was acknowledged in 7% (3/45) of the studies [72,73,79].

### DTs in the Included Studies

#### Overview

Among the 45 primary studies, data related to DTs and PFMT were not extracted from 4 (9%)—1 (25%) [55] analyzed data collected from 3 previous RCTs [5,6,64], and 3 (75%) qualitative studies [46,77,80] took a broad approach without focusing on a specific technology for the delivery of PFMT. Therefore, the data and information in the following sections were derived from 91% (41/45) of the studies, some of which used the same DTs (eg, Tät, Leva, and Pen Yi Kang; Table 1).

**Table 1:** Summary of digital technologies (DTs) and their features.

Study^a^	DT	BF^b^	Mobility requirements	EB^c^	DE^d^	EF^e^	R and R^f^	Social media features	Self-monitoring	Gamification	Training or support in use of DT
Anglès-Acedo et al [37,38]	Mobile app—WOMEN UP	Yes	Internet; Bluetooth	Yes	Yes	Yes	Yes; NI^g^	NI	Yes	Yes	NI
Araujo et al [39]	Mobile app—Diário Saúde	No	Internet	NI	Yes	No	Yes; yes	No	NI	No	NI
Asklund et al [5]; Asklund and Samuelsson [66]; Nyström et al [73]; Rygh et al [63]; Samuelsson et al [50]	Mobile app—Tät	No	Internet	Yes	No^h^	Yes	Yes; yes	No	Yes	No	Yes
Wadensten et al [64]	Mobile app—Tät II	No	Internet	Yes	No	Yes	Yes; yes	NI	Yes	No	Yes
Bokne et al [41]	Internet-based program—Tät	No	Internet	Yes	No	Yes	No; no	No	NI	No	NI
Firet et al [56]	Internet-based program—Tät	No	Internet	Yes	Yes	Yes	NI; yes	NI	Yes	No	Yes
Sjöström et al [6]	Internet-based program—Tät	No	Internet	NI	No	Yes	No; yes	No	Yes	No	Yes
Barbato et al [40]	Internet-based program	No	Internet	NI	No	Yes	No; no	No	No	No	No
Campbell et al [42]	Mobile app—Squeezy App	No	Internet; Bluetooth	NI	Yes	NI	No; yes	No	Yes	No	NI
Robson [74]	Mobile app—Squeezy App	No	Internet	Yes	No	Yes	Yes; yes	NI	Yes	No	NI
Carrión Pérez et al [43]	Telerehabilitation device and vaginal probe	Yes	Internet; Bluetooth	NI	Yes	NI	NI; NI	No	NI	No	Yes
Coggins et al [44]	Mobile app and vaginal device—Elvie	Yes	Bluetooth	NI	Yes	NI	Yes; yes	NI	NI	No	NI
Conlan et al [45]	Telehealth	No	Internet	NA	No	No	No; no	No	No	No	NI
Cornelius [71]	Mobile app and vaginal probe—PeriCoach	Yes	Bluetooth; internet	NI	Yes	NI	Yes; yes	NI	Yes	No	NI
Shelly [76]	Mobile app and vaginal probe—PeriCoach	Yes	Bluetooth; internet	NI	Yes	NI	Yes; NI	NI	Yes	No	Yes
Smith [53]	Mobile app and vaginal probe—PeriCoach	Yes	Bluetooth; internet	NI	NI	NI	NI; NI	NI	NI	No	Yes
Dufour et al [67]	Mobile app and vaginal device—iBall	Yes	Bluetooth	NI	Yes	NI	Yes; NI	Yes	Yes	Yes	Yes
Goode et al [58]	Web-based—MyHealtheBladder	No	Internet	Yes	Yes	Yes	Yes; yes	NI	Yes	No	NI
Han et al [72]	Mobile app—Bwom	No	Internet	NI	NI	Yes	NI; NI	NI	NI	NI	NI
Hui et al [59]	Telemedicine continence program (videoconferencing)	No	Internet	NI	No	Yes	No; no	No	No	No	Yes
Jaffar et al [60]	Mobile app—KEPT^i^-app	No	Internet	Yes	Yes	Yes	Yes; yes	NI	Yes	NI	Yes
Kinouchi and Ohashi [68]	Smartphone-based reminder system	No	Internet	NI	Yes	No	NI; yes	NI	NI	No	Yes
Fischer Blosfield et al [57]	Mobile app—iPelvis	No	Internet	Yes	Yes	Yes	Yes; yes	Yes	Yes	Yes	Yes
Moossdorff-Steinhauser et al [52]	Mobile app—iPelvis	No	Internet	NI	NI	Yes	Yes; yes	NI	Yes	NI	NI
Li et al [69]	Mobile app and audio guidance—Pen Yi Kang	No	Internet	Yes	Yes	NI	NI; NI	NI	NI	NI	Yes
Wang et al [51]	Mobile app and audio guidance—Pen Yi Kang	No	Internet; Bluetooth	Yes	Yes	NI	Yes; NI	NI	NI	No	Yes
Li et al [47]	Mobile app—UIW^j^	No	Internet	Yes	Yes	Yes	Yes; yes	NI	Yes	NI	Yes
Loohuis et al [61]; Wessels et al [65]	Mobile app—UrinControl	No	Internet	Yes	Yes	Yes	NI; yes	NI	Yes	No	Yes^k^
Moretti [36]	Mobile app, vaginal probe, and surface electrodes—MyoPelvic	Yes	Bluetooth	Yes	Yes	NI	NI; NI	NI	Yes	Yes	Yes
Pedofsky et al [48]	Mobile app and intravaginal pressure sensor array—FemFit	Yes	Bluetooth	NA	NI	Yes	NI; NI	NI	NI	Yes	No
Pla et al [49]	Mobile app and vaginal device—Birdi	Yes	Bluetooth; internet	NI	Yes	NI	Yes; NI	NI	Yes	No	Yes
Pulliam et al [62]	Mobile app and vaginal insert—Leva Pelvic Digital Health System	Yes	Bluetooth	NI	Yes	NI	Yes; NI	NI	Yes	No	Yes
Weinstein et al [54]	Mobile app and vaginal insert—Leva Pelvic Digital Health System	Yes	Bluetooth; internet	NI	Yes	Yes	Yes; NI	NI	NI	No	Yes
Saboia et al [75]	Mobile app—Continence App	No	Internet	Yes	Yes	Yes	Yes; yes	NI	Yes	NI	NI
von Au et al [70]	Mobile app—Pelvina	No	Internet	NI	Yes	Yes	NI; NI	NI	NI	No	NI

^a^Studies are ordered alphabetically but grouped by app where relevant.

^b^BF: biofeedback.

^c^EB: evidence base (provide some evidence base or previous testing of the DTs).

^d^DE: data extraction (capacity to extract data).

^e^EF: educational features.

^f^R and R: reinforcements and reminders.

^g^NI: not indicated.

^h^With the exception of the study by Asklund and Samuelsson [66], in which women had the choice to use the statistics function.

^i^KEPT: Kegel Exercise Pregnancy Training.

^j^UIW: Urinary Incontinence for Women.

^k^Not indicated in the study by Wessels et al [65].

#### Types of DTs (Consensus on Exercise Reporting Template for PFMT Item 1)

A total of 28 types of DTs were used across the 41 studies. Just over 40% (12/28, 43%) of these were solely mobile apps, 4% (1/28) trialed a smartphone-based messaging system, 11% (3/28) were internet-based programs, and 7% (2/28) were dedicated to videoconferencing. A total of 32% (9/28) of the technologies involved the use of a portable vaginal biofeedback or accelerometer-based device [36-38,43,44,48,49,53,54,62,67,​^71^,^76^] that provided real-time feedback transmitted via Bluetooth to a mobile app or, in one instance, to a computer application [43]. In addition to the vaginal device, electromyographic data were integrated from surface electrodes attached to the abdominal muscles [37,38] or PFMs [36].

#### Evidence Base or Previous Testing of DTs

A total of 54% (22/41) of the studies provided some evidence base for or testing of the DTs [5,41,50,51,56,58,61,63-66,69,​^73^,^74^], which had been either undertaken in the development stage or as an iterative process (eg, Tät) or was one of the specific purposes of the study [11,36-38,47,48,60,75]. Some studies (4/41, 10%) implemented a design framework (eg, the Reach, Effectiveness, Adoption, Implementation, and Maintenance framework [47] or the Fit Between Individuals, Task, and Technology framework [56]) in their trials. The development of the technologies generally involved collaboration, testing, and input from IT experts (such as hardware or software engineers) and HCPs or researchers with relevant clinical expertise (eg, obstetricians, women’s health physiotherapists, and nurses) and feedback from women, the end users of the product. One app (1/12, 8%) was developed based on 12 key variables identified through a systematic review of the literature [57].

#### Capacity to Extract Data

Approximately 60% of the studies (25/41, 61%) reported the capacity to extract data to monitor women’s progress; approximately 30% did not (12/41, 29%), and this was not indicated in 10% (4/41) of the studies. Data were extracted directly from the DTs in 20% (5/25) [39,43,49,61,65], and in 1 (4%) of the 25 studies, data could be emailed to the researchers via the app [36]. In other studies (19/25, 76%), data were transmitted from an app and stored or accessed on an associated web platform or server [37,38,42,44,47,56-58,68-71,75,76] or uploaded from the app to a cloud-based storage system [51,54,60,62,67]. Of the suite of studies that used Tät, 11% (1/9) indicated the collection of user statistics from the internet-based program [56], and 22% (2/9) reported that women could voluntarily choose to use the statistics function and submit their user statistics at follow-up [5,64].

#### Educational Features (Consensus on Exercise Reporting Template for PFMT Item 10)

Educational information was incorporated into 61% (25/41) of the studies, with most including a combination of topics such as education about the anatomy and function of the PFMs, PFMT, stress UI, and related lifestyle advice (eg, weight management, physical activity, and fluid management). A total of 2% (1/41) of the studies provided holistic advice on breathing, posture, and movement [40] and used videos to deliver this information, an approach also adopted by another 7% (3/41) of the studies [57,72,81], with another incorporating audio fragments [56]. In a videoconferencing study, education was provided by a nurse specialist across a series of talks rather than being integrated into the technology itself [59]. Of the studies that did not incorporate education, 6% (1/16) involved telerehabilitation [43], and 12% (2/16) were mobile apps [39,68], with 6% (1/16) solely using a smartphone reminder system [68].

#### Reinforcements, Reminders, and Self-monitoring (Consensus on Exercise Reporting Template for PFMT Items 5 and 6)

A variety of reinforcements were used across 59% (24/41) of the studies, but the most common was the provision of visual (eg, graphics) or audiovisual feedback to guide women on the performance of PFMT, with the inclusion of voice [52,57] or sound [47] commands or accompanying music [39]. Goode et al [58] included storytelling; another study had an exercise module with a timer and score board [60].

In just over 50% (21/41, 51%) of the studies, reminder systems were incorporated into or complemented the DTs. In most cases (13/21, 62%), the reminders were customizable and were set by the women; in 24% (5/21) of the studies, push notifications were sent by the researcher or HCPs [21,47,52,60,68], and in 14% (3/21) of the studies, women were emailed a reminder (internet-based programs) [6,56,58].

Self-monitoring was a feature in 61% (25/41) of the studies. Apps commonly enabled tracking of exercise progress by women, including a statistical function (eg, Tät) or graphs or the capacity to record exercise adherence over time (eg, number and level of exercises). This function was also available through a web portal [37,38,76], or training diaries were completed and sent via email [6]. Some technologies also included a bladder diary [58,60,71] to monitor urinary symptoms.

#### Social Media and Gamification

In total, 7% (2/28) of the DTs had the capacity for social media forums: the iPelvis, which included a website and Facebook page [11,57], and the iBall [67], which enabled women to connect with others in a web-based community (but it was disabled for the purpose of the study, as it was only available in Chinese).

A total of 21% (6/28) of the DTs incorporated gamification [36-38,48,57,67], which, with 1 exception [57], was used in conjunction with biofeedback. Descriptions of gamification included “serious games” [37,38], games or activities (eg, weight lifting room and flying arena) [67], and gaming and virtual reality mediated by a comic character [11,57] or a cyclist [36] with built-in scoring systems.

#### Technical Support

A total of 32% (13/41) of the studies offered instructions (eg, handouts and instructions via email) on how to download and install the app or use and effectively care for the equipment (eg, vaginal probes) [5,47,49,51,53,54,57,60,61,64,67-69].

Follow-up technical support was offered in 15% (6/41) of the studies by a research assistant [6,47,51,54,56,60] using encrypted email or via the app. A total of 10% (4/41) of the studies included in-person sessions with supervision or testing of the technology [36,43,62,76] by physiotherapists [36,43,76] or an unspecified individual [62].

### PFMT in the Included Studies

#### Evidence Base

Just over half (21/41, 51%) of the studies provided some evidence base for the PFMT program that was being delivered via the DTs; in the remaining studies, this was not indicated or was unclear. Evidence for PFMT varied, ranging from existing programs tested in RCTs, including the seminal publication by Bø et al [119] and others later (eg, the *Group Rehabilitation Or IndividUal Physiotherapy for Urinary Incontinence in Aging Women* [GROUP] trial [120]), to expert opinion [121], guidelines (eg, the National Institute for Health and Care Excellence) [122], and the Dutch clinical practice guidelines for the physiotherapy management of stress UI [123], with enhancements made based on feedback from clinicians, women, and researchers (eg, Tät).

#### Delivery of PFMT (Consensus on Exercise Reporting Template for PFMT Items 1, 2, 3, 4, and 12)

On the whole, women engaged with the DTs at home on an individual basis, with 22% (9/41) of the studies including exercise both at home and in a clinical setting [39,42,43,51,52,57,62,76] or community center [59] (Table 2; please note that, in some cases, the same DT was used across multiple studies). In 10% (4/41) of the studies, women also attended supervised group sessions once a week to undertake PFMT via teleconferencing [59], in person [52,57] with a maximum of 4 women per group [52], or specific to one of the study arms (app plus physiotherapy group) [57]. In the study by Pla et al [49], Skype was the medium for supervision of group-based hypopressive abdominal exercises 3 times per week (5-9 women per group), with women also receiving monthly individual videoconferencing sessions to check progress.

In addition to the 10% (4/41) of studies that provided supervision for women in a group setting [49,52,57,59], 34% (14/41) supported women on an individual basis. Examples included confirming a PFM contraction or PFMT practice [39,51] and checking women’s adherence to the program [39]; providing a set number of supervised sessions over the duration of the program (which ranged between 1 and 12) either in person [42,76] or remotely via email [6,41], phone call [54], or videoconferencing [45]; and more intense supervision, such as five 30-minute sessions over 2 weeks [43] and daily sessions 5 days per week [62]. If women required extra support with PFMT-related content, this was offered through email [45,49,56] or the chat function on an app [54], with Anglès-Acedo et al [37,38] noting that their web platform enabled “personalised supervision.”

Details of the personnel providing supervision or support for PFMT were reported in 18 studies, with 8 (44%) referring to a physiotherapist [49,57] who was specialized in women’s or pelvic health [39,42,43,45,52,76]. In others, a nurse specialist [59], therapist [37,38], urogynecologist [61], urotherapist [6,41], general practitioner [56], trained researcher [51], trained research assistant [62], or trained study staff member [54] provided supervision or support.

**Table 2:** Summary of pelvic floor muscle training (PFMT) delivery and content.

Study^a^	DT^b^	PFMT evidence base	Individual or group PFMT and setting	Supervision of PFMT and qualifications	Confirmation of voluntary PFM^c^ contraction	PFMT parameters	Duration of program
Anglès-Acedo et al [37,38]	Mobile app—WOMEN UP	NI^d^	Individual; home	Web platform; therapist	Biofeedback	NI	3 months
Araujo et al [39]	Mobile app—Diário Saúde	NI	Individual; home and clinic	In person, monthly; specialist women’s health physiotherapist	Digital assessment; specialist physiotherapist	8-second hold, 8-second relaxation followed by 3 phasic contractions 8 times, 2 times per day (sitting, lying down, or standing)	3 months
Asklund et al [5]; Asklund and Samuelsson^e^ [66]; Nyström et al^f^ [73]; Rygh et al^f^ [63]; Samuelsson et al^f^ [50]	Mobile app—Tät	Yes	Individual; home	No; —^g^	No	Progressive PFMT (6 basic and 6 advanced levels), different combinations and repetitions of PFM contractions (strength and endurance, quick contractions, and the “knack”); advanced phase incorporates different positions (standing, lifting, and walking); strengthening: from a 5-second hold, 5-second relaxation 2 times (basic) to a 7-second hold, 7-second relaxation 40 times (advanced); endurance: from a 14-second hold once (basic) to a 59-second hold, 59-second relaxation 2 times (advanced); quick: from a 3-second hold, 3-second relaxation 5 times (end of the basic phase) to a 3-second hold, 3-second relaxation 20 times (advanced) 3 times daily	3 months
Wadensten et al [64]	Mobile app—Tät II	Yes	Individual; home	No; —	No	Progressive, 4 different PFM exercises are included across 8 modules based on the Tät [5], from 3 times per day for 2 minutes (module 1) to 3 times per day for 3-4 minutes (module 4) and 3 times per day for 12 minutes (module 8)	15 weeks
Bokne et al [41]; Sjöström et al [6]	Internet-based program—Tät	Yes	Individual; home	Email once per week plus support as needed; urotherapist	No	Progressive, tailored (in part) program with 8 levels, including the “knack”; strength: hold maximal contractions for 8 seconds, 8-10 repetitions, 3 times per day; endurance: hold submaximal contractions for 15-90 seconds, 1 repetition, 3 times per day; quick contractions: hold for 3 seconds, 8-10 repetitions, 2-3 times per day	3 months
Firet et al [56]	Internet-based program—Tät	Yes	Individual; home	Email support as needed; GP^h^ in training or researcher	No	Progressive, 4 different PFM exercises are included across 8 modules based on the Tät [5], from 3 times per day for 2 minutes (module 1) to 3 times per day for 3-4 minutes (module 4) and 3 times per day for 12 minutes (module 8)	3 months
Barbato et al [40]	Internet-based program	NI	Individual; home	No; —	No	“Self-paced” PFMT, 10-15 minutes daily	3 weeks
Campbell et al [42]	Mobile app—Squeezy App	NI	Individual; home and clinic	In person, ≤7 appointments (45-60 minutes) over 6 months depending on women’s needs; specialist pelvic health physiotherapist	Digital assessment (crook lying) and specialist physiotherapist; biofeedback (standing)	Progressive, tailored PFMT (in different functional positions) for strength, power, endurance, and relaxation, and the “knack”; no specific details of the PFMT program	Phase 2: 6 months^i^
Robson [74]	Mobile app—Squeezy App	NI	Individual; home	—	—	NI	Survey, open for 3 months
Carrión Pérez et al [43]	Telerehabilitation device and vaginal probe	NI	Individual; home and clinic	In person, 5 times for 30 minutes over 2 weeks plus monthly follow-up; pelvic floor expert physiotherapist	Biofeedback	PFMT: five 30-minute sessions in the clinic (over 2 weeks) plus home exercise program; daily	3 months
Coggins et al [44]	Mobile app and vaginal device—Elvie	NI	Individual; home	No	Biofeedback	NI	NI
Conlan et al [45]	Telehealth	NI	Individual; home	In person, initial 1-hour session plus email support over 6 weeks^j^; continence physiotherapist	NI	Individualized PFMT	6 weeks
Cornelius [71]	Mobile app and vaginal probe—PeriCoach	NI	Individual; home	NI; pelvic floor clinicians (for some participants)	Digital palpation^k^; biofeedback	Dosage^k^: contraction, relaxation 5 times for 5 seconds, 10 repetitions, 4 times per day, 5 times per week	8 weeks
Shelly [76]	Mobile app and vaginal probe—PeriCoach	NI	Individual; home and clinic	In person, 6 sessions over 8 weeks; pelvic floor physiotherapy specialist	Digital assessment and specialist physiotherapist; biofeedback	Progressive, tailored, starting with contraction, relaxation 3 times for 8 seconds, 8 repetitions (20-25 repetitions per day; week 1); 5 times for 7 seconds, 8 repetitions (40-50 repetitions per day; week 2); 6 times for 3 seconds, 15 repetitions (week 5); 10 times for 3 seconds, 15 repetitions (week 8); supine, then standing; functional training with forward bending and during ADLs^l^ 2 times per day, 5 times per week	8 weeks
Smith [53]	Mobile app and vaginal probe—PeriCoach	NI	Individual; NI	NI; NI	NI	NI	20 weeks
Dufour et al [67]	Mobile app and vaginal device—iBall	Yes	Individual; home	No; —	Digital palpation; specialist pelvic health practitioner	3 times, 10 sets of 10 exercises 3-4 times per week	16 weeks
Goode et al [58]	Web-based—MyHealtheBladder	Yes	Individual; home	No; —	No	Progressive, from contraction, relaxation 2 times for 4 seconds (week 1) to 5 times for 5 seconds (week 4), 9 times for 9 seconds, and 10 times for 10 seconds (week 8) plus bladder control strategies	8 weeks
Han et al [72]	Mobile app—Bwom	NI	Individual; home	No; N/A^m^	No	Progressive, “personalized” exercise plans, each with 6-12 exercises, with a new exercise each week	2 weeks
Hui et al [59]	Videoconferencing	NI	Individual and group; home and community center	Weekly videoconferencing; nurse specialist assisted by a research assistant (registered nurse)	Digital assessment and nurse specialist; biofeedback	1 videoconferencing session per week	8 weeks
Jaffar et al [60]	Mobile app—KEPT^n^-app	Yes	Individual; home and clinic	No; —	No	Progressive, 3 training skills and modes (different positions): beginner (2-second hold), intermediate (6-second hold), and advanced (10-second hold); 10 repetitions, 3 times per day; adherence phase: once they can perform PFMT confidently, maintain 10 cycles, 3 times per day	At least 16 weeks^o^
Kinouchi and Ohashi [68]	Smartphone-based messaging system	Yes	Individual; home	No; —	No	Hold 3-6 seconds, 3 sets of 6 contractions per day; different positions (standing, bent-knee lying, and 4-point kneeling)	8 weeks
Fischer Blosfield et al [57]	Mobile app—iPelvis	Yes	Individual and group; home and clinic (depending on study group allocation)^p^	12 sessions once a week, in person, in a group; physiotherapist	All participants had “physical examination”; women who had difficulty contracting PFM had a vaginal examination, with instruction	App+physiotherapy: PFMT in a group once per week plus app at home App only: PFMT at home. Progressive and tailored, including strength, explosive strength, endurance, timing, precontraction, exhaustibility, coordination, and functional exercises (eg, sneezing and coughing); PFM contraction and relaxation in different positions, situations, and activities, 6 phases, each with a 15-day duration	3 months^p^
Moossdorff-Steinhauser et al [52]	Mobile app—iPelvis (in conjunction with the Motherfit program)	Yes	Individual and group; home and clinic	8 sessions in person (60 minutes) in a group (maximum of 4 women); specialist pelvic physiotherapist	Observation and digital assessment, supine; pelvic specialist physiotherapist	Progressive group program, including strength and endurance, speed, and functional exercises, and the “knack”; NI if the home program was the same; build up to 8-12 contractions, 6-8–second hold plus 3-4 fast contractions; strength and endurance: 3 times per day, daily (minimum of 3-4 times week); different positions (lying down, sitting, kneeling, and standing); after 6 months of training: maintenance 2 times per week; speed: fast repetitions, build up to 10 sets of 3 quick contractions and 10 sets of 5 quick contractions 3 times per day	8 weeks, continuing past 6 months of home training
Li et al [69]	Mobile app and audio guidance—Pen Yi Kang	NI	Individual; home	No; —	Digital assessment; experienced physiotherapist	NI	6 weeks
Wang et al [51]	Mobile app and audio guidance—Pen Yi Kang	NI	Individual; home	In-person initial 45-minute session plus phone contact once a month; trained researcher	Digital palpation; surface EMG^q^, supine, and hips and knees bent	Progressive, different positions (sitting, standing, and lying down); 3-second hold, 2-6–second relaxation for 15 minutes, 2 times per day or 150 contractions per day	3 months
Li et al [47]	Mobile app—UIW^r^	Yes	Individual; home	No; —	Perineum palpation, supine, and surface EMG in lithotomy position; experienced obstetrician	Adapted from Tät [5]; progressive, 2 basic and 4 advanced levels, including different combinations and repetitions of 4 commonly used contraction types: test contraction, strength contraction, endurance contraction, and quick contraction; up to each woman to determine use (frequency and duration)	8 weeks
Loohuis et al [61]	Mobile app—URinControl	Yes	Individual; home	No; —	Assessed according to the ICS^s^; urogynecologist	Progressive program, directed to appropriate part of the app to start training; no further information provided	4 months
Wessels et al [65]	Mobile app—URinControl	Yes	Individual; home	No; —	No	Progressive program, directed to appropriate part of the app to start training; no further information provided	—
Moretti [36]	Mobile app, vaginal probe, and surface electrodes—MyoPelvic	Yes	—	—	Biofeedback, maximal voluntary contraction, and supine; researcher	Phasic fibers: contract <4 seconds, relax for twice the duration of the contraction, 12 repetitions maximum (as dictated by the game); tonic (slow) fibers: contract 4-10 seconds, relax for the same duration, 12 repetitions maximum (as dictated by the game); 1-2–minute rest between games recommended but not enforced; muscle coordination training (not specified)	—
Pedofsky et al [48]	Mobile app and intravaginal pressure sensor array—FemFit	Yes	—	—	—	Progressive, graduated exercise; no further information provided	12 weeks
Pla et al [49]	Mobile app and vaginal device—Birdi	NI	Individual and group; home	Videoconferencing, 2 initial individual sessions 3 times per week in a group and monthly individual session plus email or phone support; physiotherapist	Measured by the device	Daily PFMT, tailored	2 months
Pulliam et al [62]	Mobile app and vaginal insert—Leva Pelvic Digital Health System	NI	Individual; home and clinic	In person, once a day, 5 times per week over 6 weeks; trained research assistant	Accelerometer-based system	15-second PFM contraction 5 times, 15-second relaxation, 2 times per day in standing position	6 weeks
Weinstein et al [54]	Mobile app and vaginal insert—Leva Pelvic Digital Health System	NI	Individual; home	3 phone calls in first 2 weeks plus support via the chat function; trained study staff	No	5 cycles of squeeze and lift, and 15 seconds of rest for 15 seconds each, 2.5 minutes 3 times per day	8 weeks
Saboia et al [75]	Mobile app—Continence App	Yes	—	—	—	NI	12 weeks
von Au et al [70]	Mobile app—Pelvina	NI	Individual; home	NI; NI	No	NI	Survey, open for approximately 1 year

^a^Studies are ordered alphabetically by first author but grouped by app where relevant.

^b^DT: digital technology.

^c^PFM: pelvic floor muscle.

^d^NI: not indicated.

^e^Survey, open for 10 months.

^f^3 months of app use.

^g^No information provided.

^h^GP: general practitioner.

^i^3 phases to this study.

^j^None of the participants used the email option.

^k^As reported in the study by Starr et al [83].

^l^ADL: activities of daily living.

^m^N/A: not applicable.

^n^KEPT: Kegel Exercise Pregnancy Training.

^o^Start at 28-week gestation until delivery at 36 weeks and continue PFMT until 8 weeks post partum.

^p^6-month program=12 phases as per Latorre et al [11].

^q^EMG: electromyography.

^r^UIW: Urinary Incontinence for Women.

^s^ICS: International Continence Society.

#### Content of PFMT (Consensus on Exercise Reporting Template for PFMT Items 7, 8, 9, 13, 14, and 15)

Confirmation of a voluntary PFM contraction was undertaken in just under half (19/41, 46%) of the studies and was either not included in the study design in 41% (17/41) of the studies or not indicated or appropriate (5/41, 12%). Confirmation was obtained through digital assessment [39,57,61,69], which was also used to teach a correct contraction or relaxation of the PFM [52,67]; biofeedback or the digital device [36-38,43,44,49,62]; or a combination of both [42,47,51,59,71,76]. Digital assessment was undertaken by a specialized HCP, although it was not indicated who performed this procedure in 7% (3/41) of the studies [51,57,71]. In the RCTs that used digital assessment, this was provided to all women across all the study groups.

A total of 49% (20/41) of the studies offered PFMT programs that were progressive in terms of content or position. Although most programs were generic, 10% (2/20) were tailored to the individual by provision of supervision [49,76], and another 15% (3/20) provided some indication or tailoring of the starting level for PFMT [61,64,72].

The details of the PFMT content were not indicated in 34% (14/41) of the studies, whereas the rest used a combination of strength, endurance, and power exercises; relaxation; or a combination of these. Direct PFMT, together with functional PFMT (eg, the “knack”), was included in 29% (12/41) of the studies [5,6,41,42,50,52,57,63,64,73,76,78], and it is likely to have been integrated into another 5% (2/41) of the studies that were based on the Tät [47,56].

Of the 19 studies that provided details about the prescribed dose, 14 (74%) recommended PFMT 3 times a day [5,6,41,47,50,52,54,56,60,63,64,68,73,78], 4 (21%) recommended PFMT twice a day [39,51,62,76], and 1 (5%) recommended it 4 times a day [71]. The most common program duration was 3 months (17/41, 41% of the studies) [5,6,37-39,41,43,50,51,56,57,63,66,73-75,84], followed by 2 months [47,49,52,54,58,59,68,71,76], with others spanning 2 to 6 weeks [40,45,62,69,72] or >15 weeks up to 6 months [42,53,60,61,64,67] to 1 year [70]. It should be noted that women in the study by Moossdorff-Steinhauser et al [52] continued exercising at home for at least 6 months after the end of the 8-week group exercise PFMT, and the 16 weeks specified by Jaffar et al [60] were the minimum program duration.

#### Adverse Events (Consensus on Exercise Reporting Template for PFMT Item 11)

A total of 20% (8/41) of the studies documented adverse events [6,36,37,43,52,54,62,64]. Adverse effects were reported in 12% (5/41) of the studies, none of which were deemed serious. Some examples include vaginal discomfort, infection, or allergic reactions related to the use of the vaginal device [37,43]; lower abdominal pain related to PFMT [6]; and increased spontaneous urine leakage [64].

#### Treatment Fidelity (Consensus on Exercise Reporting Template for PFMT Item 16)

A total of 5% (2/41) of the studies, both of which were protocols [47,60], assessed the implementation of the intervention. The methods used included monitoring participant activity and training time through the app [60], tracking technical support provided, consultation support, and reminders sent to women who had not used the app over the previous week [47].

### UI Outcomes

UI outcomes following DTs were presented in 56% (23/41) of the studies (Table S1 in Multimedia Appendix 4 [5,6,39-41,43-45,49-51,53,57-59,61-64,67,68,73,76]), with improvements reported across measures in all but 1 study (within groups; 22/23, 96% [68]). All studies (23/23, 100%) analyzed changes in UI symptoms and severity or general or UI-specific QoL as measured through self-reported questionnaires, UI episode frequency, pad weight tests, or UI aid use.

### Adherence to the Program

A total of 61% (25/41) of the studies indicated methods for evaluating adherence, with some (9/25, 36%) using more than one approach (Table S2 in Multimedia Appendix 4 [5,6,37,39,41-44,47,50-52,54,56,58,60,61,63,64,67-69,71,73,74]). In total, 72% (18/25) of the studies gathered data from the DTs themselves [5,37,39,42,43,47,51,52,54,56,58,60,61,64,67-69,​^79^], 48% (12/25) used self-report via email [6] or a web- or app-based questionnaire [5,41,44,50-52,61,63,64,74,79], and 4% (1/26) included in-person appointments [42].

A total of 68% (17/25) of the studies provided data following completion of the program. Women in 76.4% (13/17, 50%) of these studies provided a self-report of adherence to the prescribed PFMT, which was measured and reported in a variety of ways—visual analogue scale [39], validated questionnaire to assess efficacy [51], number of exercises completed over specified time points (eg, in the last month or last week, daily, weekly, or monthly) [5,41,44,50,58,63,74,79], or percentage of women who performed PFMT [37,68] or adhered to the program [58] or some of the program [85]. Self-reported daily PFMT for women using DTs ranged from 23.4% to 41% over 3 months [5,63].

Performance of PFMT was also captured via the DTs in some studies (9/25, 36%), including measures of how often the app was used (eg, never, once a week, and >3 times per week) [50,61,63,64,69], mean number of exercises performed per day (1.6 [5]), median number of days PFMT was performed per week (4.9 [43]), and percentage of women who completed at least 75% of the study requirements for the program (14.4% [85]).

Two studies compared adherence to the DTs with a control group. Adherence was significantly higher (*P*<.001) in the group using the DTs at 1, 2, and 3 months [39], but there was no difference (*P*=.40) reported between the groups in the study by Carrión Pérez et al [43].

### Satisfaction With DTs and Outcomes

A total of 63% (17/41) of the studies considered satisfaction with the DTs, including reporting on experiences with specific aspects (eg, PFMT, exercise logs, reminder features, and ease of accessing videos or instructions) [5,36,37,39,40,44,45,​^52^,^54^,^59^,^60^,^62^,^64^,^67^,^72^,^74^,^75^] (Table S2 in Multimedia Appendix 4). Although a range of different outcome measures was used, it appears that most participants were satisfied with the DTs and would recommend them to others. An exception was the study by Dufour et al [67], in which only 18.2% (2/11) of the women would recommend the mHealth device or consider using it again; however, this response increased to 63.6% (7/11) if the device were to be modified.

Satisfaction with the program as a whole, or self-reported improvement, was reported in 27% (11/41) of the studies [5,6,40,43,44,49,52,54,58,64,71]. Responses varied with respect to overall satisfaction, ranging from not satisfied (6% in the study by Goode et al [58] and 33% in the study by Wadensten et al [64]) to somewhat satisfied (75% in the study by Goode et al [58]) and completely satisfied and symptom-free (7% in the study by Wadensten et al [64]). Although satisfaction in the study by Sjöström et al [6] was higher in the intervention (app) group at 4 months, there was no significant difference at the 1- and 2-year follow-ups; no difference between groups was reported by Carrión Pérez et al [43]. Self-reported improvement in symptoms was variable, with <25% of the women in 18% (2/11) of the studies [40,44] reporting that they were much or very much better (10.3%-23.5%) with respect to symptoms, but more women (55.7%) reported the same for self-perceived improvement in PFM strength [5].

### Qualitative Synthesis: Experiences of Women and HCPs

The summary data from the completed qualitative or mixed methods studies (11/45, 24%) are presented in Multimedia Appendix 5 [31,38,42,46-48,65,67,77,78,80,86,87]. In almost all the studies, the same factors were presented as both facilitators and barriers. This demonstrates that preference for or against any given DT may be related to the individual and that personal preferences can change over time.

The results demonstrated some overarching facilitators. Participants liked the anonymity that DTs provide for the treatment of UI symptoms [31,38,46,65,86,87]. Several studies (6/11, 55%) discussed that UI is still considered a socially “taboo subject,” a topic that women can find difficult to acknowledge to themselves, let alone discuss with an HCP [31,65,77,80,86,87]. Being judged or feeling embarrassed to discuss UI was a common finding, and the opportunity to access DTs provided women with a viable, accessible, and potentially less time-consuming alternative means of seeking support [31,38,46,48,65,77,78,80,86,87]. Furthermore, the ability to use an app in the convenience of their own environment facilitated empowerment, confidence, and self-efficacy regarding the ability to manage their UI symptoms with PFMT exercises [31,38,46,48,65,77,78,86,87].

All studies (11/11, 100%) demonstrated that the knowledge content across the various apps was helpful. Knowledge included gaining a better understanding of the causes of UI, where PFMs are and what is their function, and the fact that UI is a common problem. In total, 18% (2/11) of the studies [65,87] reported that women felt less isolated after learning about the prevalence of UI.

DTs were considered successful by participants if an improvement in UI symptoms was observed [31,67,78,86,87]. Participants were reported to be more adherent when UI symptoms were more severe [31,86,87] and less adherent to PFMT as UI symptoms improved [31,65,78,86,87]. However, other personal and technological factors also influenced adherence to the various PFMT programs and, thus, UI symptom outcomes. These included the needs of other family members, especially for new mothers; concomitant health issues; and other life events [31,65,78,80,86,87].

Establishing a routine; the use of reminders, journals, and diaries; and family support went some way toward mitigating these barriers [31,46,77,78,86,87]. Although some HCPs expressed concerns about the ability of older women to use DTs [80], the studies in this review suggest that the competing time pressures experienced by women with young families, especially if they were working, were more of a barrier [31,65,67,87]. Culture was not discussed in any of the studies, so it is unclear whether the same facilitators and barriers apply across all cultures and ethnic groups.

Another common finding across the studies was concern about the ability to “correctly” contract the PFMs. Although the concept of an internal exam to determine a “correct” contraction was not always appealing [65], being unsure of whether the exercises were being performed correctly was a barrier to adherence [31,46,65,77,78,80,86,87]. Consequently, several studies (8/11, 73%) [31,46,48,65,77,78,80,87] suggested that engagement with HCPs, perhaps for an initial assessment and then for progression at a later point in time, was an important facilitator. Other studies found that HCP consultations were required to support adherence and provide encouragement and progression of PFMT in addition to the benefits of DTs [31,86]. Both consultations with HCPs and DTs (if from a recognized institution, such as a university) reassured participants that the information they received and the PFMT program they were trying were from a credible source [31,77].

As per the results in Multimedia Appendix 5, technology that was easy to set up, insert (if applicable), comfortable, and portable was more acceptable to participants [31,38,46,48,67,77,78,87].

## Discussion

### Principal Findings

This systematic scoping review was undertaken to explore the range and features of DTs available for managing UI. Specifically, we sought to determine whether the PFMT embedded in DTs follows best-practice guidelines, is designed for women at specific stages in life, and considers cultural contexts and the experiences of women and other relevant stakeholders.

It is evident that the medium of DT for the conservative management of UI is prevalent and continually expanding, with rapid growth apparent particularly over the last 10 years. In total, 89 studies were included in this scoping review—51% (45/89) were primary studies and 49% (44/89) supplementary papers—which is larger than the number (between 3 and 10 papers) included in several recent narrative and systematic reviews in this field [7,8,18-21,124,125]. This difference likely reflects variations in inclusion and exclusion criteria, which in this study were intentionally broad so as to encompass a range of sources, including qualitative research.

The WHO global strategy on digital health stipulates that DTs should be “people-centred, trust-based, evidence-based, effective, efficient, sustainable, inclusive, equitable and contextualised” [126]. In terms of the evidence-based dimension, it is encouraging that over half of the DTs (22/41, 54%) were developed based on evidential research or testing. The means of achieving this varied across the studies, but most adopted an iterative process of continuous testing, implementation, and refinement. IT input is obviously integral to the development of DTs, but importantly, a number of studies in this review took a user-centered approach by seeking the opinions of women with or without UI and, in some cases, HCPs who may be involved in a woman’s care. Considering users’ opinions, needs, and expectations at all stages of DT design is not only endorsed by the WHO [126] but is also vital in optimizing the usability and acceptability of the DTs and their adaptation to ensure effectiveness in outcomes [127]. Some studies (4/41, 10%) adopted a theoretical user-centered framework to guide the design of the DTs [47,48,56,60], and standardization and use of such frameworks by future developers will assist in continued improvements in the quality of DT apps specifically for PFMT, which could ultimately enhance the conservative management of UI.

Free and commercial PFMT apps are readily available for download from app stores, but only some are clinically sound from a PFMT perspective [128], with many lacking in terms of accuracy, content, quality, and functionality [10,128-130]. Just over half (21/41, 51%) of studies documented that the PFMT programs were drawn or adapted from a known evidence base, which suggests that they are in line with the recommendations of PFM exercise theory that lead to improvements in UI symptoms [122,131]. However, there was a large variation in the PFMT reported, including the type of exercise, dose, frequency, progression, and supervision, and some PFMT details were often incompletely reported (particularly in abstracts, which is to be expected). In addition to details about PFMT, other items in the Consensus on Exercise Reporting Template for PFMT guidelines [28] were also inconsistently adopted across the studies—less than half incorporated confirmation of a voluntary PFM contraction (19/41, 46%) or reported on adverse events (8/41, 20%) or treatment fidelity (2/41, 5%), whereas just over half used reminder systems available with the DTs (21/41, 51%). From a technological perspective, some of these items, such as reminders (eg, individualized push notifications), and other features, such as social media and gamification (used in 2/28, 7% and 6/28, 21% of the studies, respectively), are suggested to be important in supporting adherence to mHealth [11,132] and are worth considering for future DTs.

As shown across a range of studies, using DTs to deliver PFMT can be effective in improving UI symptoms and QoL. In the 56% (23/41) of the studies that reported outcome measures, improvements were seen across most outcome measures for women using DTs and, in the case of comparison groups, often for those who were receiving PFMT via an alternative method (eg, pamphlet or usual care). Many of the outcome measures were self-reported, which is appropriate, as the lived experience of women is of interest. As the qualitative data show, aspects such as convenience and reduction in symptoms were of most relevance, which reinforces the need for future studies to include qualitative components to determine relevance to the primary end user. Women’s satisfaction with the program as a whole, as documented in 27% (11/41) of the studies, was variable in terms of outcome measures and data but was likely closely connected with UI outcomes. For example, in an RCT [6], the satisfaction of the women using the app was higher than that in the control group (printed PFMT) at 4 months, aligning with a significant improvement in UI symptoms; however, there was no difference between the groups at the 1- and 2-year follow-ups [88], when the effectiveness of the intervention had also waned, as had adherence to the prescribed intervention program. These findings suggest that PFMT delivered via DTs is promising as a first-line conservative management for UI, but more high-quality research, which includes long-term follow-up, is required.

There was heterogeneity in the definitions of adherence used by the studies included in this review and the methods (eg, DTs and web-based questionnaires) and measurements used to monitor this. In addition, reporting of adherence data was variable with little standardization, making comparison difficult. Among the 2 RCTs that measured adherence, in 1 (50%; 21 women), adherence was significantly better in those who used an app in the short term (up to 3 months) [39]. However, no difference was found in UI symptoms between groups, consistent with the findings of the other RCT that compared telerehabilitation and control [43]. A known problem with app use is attrition after they have been downloaded. Examples from other areas of health research suggest that approximately 20% to 25% of apps are used only once or infrequently, with use dramatically reducing to <5% over a short period (eg, 8 sessions or 15 days) [132,133]. The self-reported daily PFMT for women using DTs ranged from 24.3% to 41% over 3 months [63,79], but no long-term data were available to determine whether this followed a downward trend. There is a plethora of research that demonstrates that managing a long-term condition with regular commitment to exercise is difficult irrespective of the condition [134,135]. Therefore, factoring this typical type of human behavior into PFMT programs delivered via DT, providing reassuring statements regarding the fact that this is typical, being kind to oneself, and knowing how to start again, would be beneficial.

Other suggested benefits of using remote or app-based technologies to deliver PFMT include helping women overcome their embarrassment about seeking help for UI, improving access to health services in remote or underdeveloped areas, and enhancing cost-effectiveness [46,61,82,136]. Although using DTs in isolation may be beneficial, personal or HCP support is also recommended [11,132]. This approach aligns with best-practice guidelines for effective PFMT [122], with supervision provided to support the behavioral aspect of exercise. In this review, many studies (18/41, 44%) incorporated HCP or researcher support either synchronously (eg, in person or remotely) or asynchronously (eg, email contact), ranging from confirmation of a PFM contraction to constant monitoring of progress across the course of the program. A notable feature from the synthesis of findings from the included qualitative studies was that engagement with an HCP was an important facilitator, not only to support adherence and progression of exercises but also because women valued knowing that they were performing the PFM exercises correctly and expressed concern if they were unsure about their technique [31,46,48,65,77,78,80,86,87]. This concern is valid as inadvertently performing an incorrect PFM contraction, such as the Valsalva maneuver, could result in an increase in intra-abdominal pressure, leading to depression of the levator ani muscle and weakening of the surrounding connective tissues, which may inadvertently increase UI [137].

Interestingly, group-based supervised PFMT (either in person or remotely) was offered in 10% (4/41) of the studies [49,52,57,59]. Although results related to improvements in UI outcomes in these studies were mixed, a recent large RCT has shown that group-based PFMT is not inferior to individually supervised PFMT in older women in the treatment of UI, with both groups also undertaking a home exercise program [138]. It is known that peer support is a key strategy to help with long-term self-management as it can facilitate individual problem-solving and goal setting, which can aid with self-efficacy [139,140]. This indicates that a group-based approach to exercise likely offers further advantages to women, such as enhanced motivation to perform PFMT and reduced stigma and feelings of isolation [141]. Given the large variation in the types and levels of support and supervision currently provided for PFMT delivered via DTs, further information is needed to establish what represents best practice in terms of integrating supervision to optimize women-centered care and UI outcomes.

Culture plays a role in how women interact with DTs [15,16], perceive UI [17,142,143], and engage with PFMT and should be taken into account when designing mHealth interventions to encourage use and enhance motivation [16]. Incorporating cultural characteristics into DTs includes considering not only the user’s needs and preferences related to functionality (eg, color, typeface, and layout) but also more implicit aspects such as values, health beliefs, religion, social practices, and language [144,145]. In this scoping review, most DTs originated in high-income countries such as the United Kingdom and the United States and most likely targeted the dominant culture. This is also exemplified by the finding that only 4% (2/45) of the primary papers were written in a language other than English [35,36]. However, some apps (the Tät in particular) have been translated into a number of different languages, and research teams have also sought user input to refine them further [56,63,78], processes that are some of several different methods to enable cultural relevance [144]. The iPelvis app [11,57] explicitly incorporates culturally relevant elements, and although these may be features of other DTs included in this scoping review, they were not described. It cannot be assumed that PFMT DTs developed in one culture and translated for use in another will be successful without consulting the cultural context of the women who will use it [146], meaning that user engagement is successful in its success. Therefore, to meet the remit of inclusive and equitable DTs [126] and reach women in low- and middle-income and remote countries, more understanding is needed of what culturally related insights are required to increase the acceptability of and engagement with these technologies [146].

Many studies (28/45, 62%) did not explicitly document information related to the delivery of PFMT via DTs for women at a specific stage in life. Of those that did, most focused on pregnancy or the postpartum period, a time when UI is highly prevalent, with a risk that it could persist and become a long-term condition in some women [1]. During the childbearing years, women experience competing interests for their energy and time, such as preparing for or caring for their new baby, which means that it is vital that they receive sufficient support to adopt and maintain PFMT [67,147]. Engaging with an HCP in conjunction with using DTs was identified as an important facilitator to support PFM exercise (physical and behavioral aspects) [31,46,48,65,77,78,80,87], and there is evidence demonstrating that starting PFMT in early pregnancy may reduce the risk of UI later in pregnancy or up to 6 months post partum [4]. However, pregnant or postpartum women might not seek help from an HCP as they may feel embarrassed about their UI symptoms [148] or think that UI is a “normal” occurrence before and soon after childbirth [149]. In these instances, DTs provide a convenient tool that can support and motivate women to exercise [11,132] in the comfort of their own environment, facilitating empowerment, confidence, and self-efficacy with PFMT [31,38,46,48,65,77,78,86,87]. An additional avenue for support could be further developing and integrating social media into DTs, enabling pregnant and postpartum women to connect with each other as well as with HCPs. In general, more evidence is required to establish the acceptability, design, development, and effectiveness of PFMT DTs across various age ranges, including both adolescent and older women, to ensure that the programs meet women’s needs and circumstances. However, HCPs should have some confidence integrating DTs for PFMT into their practice as, in partnership with a clinician, this may offer women another tool in the management of UI symptoms.

### Limitations

As this scoping review included a wide range of studies and a variety of DTs, heterogeneity was evident across many study parameters, including the PFMT programs and UI outcomes, and the duration of the trials was relatively short, demonstrating the need for longer follow-up and high-quality data in this developing field of research. Biofeedback is broadly considered a DT; however, we only included studies that provided feedback to women via an app, meaning that we did not capture valuable data from trials of biofeedback that did not have this feature [150,151]. Many studies were from high-middle–income urban settings, which restricts the diversity of the target populations despite one of the benefits of mHealth being its ability to reach a range of people, including those in remote areas [61,82,136]. This review considered women with stress UI and, therefore, did not explore the impacts of PFMT DTs on other conditions or populations, such as urge UI or pelvic organ prolapse or men. As described previously, owing to the large volume of data, we were unable to implement some elements of our a priori protocol, such as synthesizing data from systematic reviews and rating the quality of the apps used in the included studies. Owing to space limitations, we were only able to present the themes most coherently relevant to the scoping review objectives, and in our synthesis, we did not consider how the quality ratings (high, fair, and poor) influenced the data.

### Conclusions

Evidence related to PFMT delivered via DTs for the conservative management of UI continues to grow exponentially. The development of DTs specifically for this purpose is increasingly based on evidential research or testing, including the exploration of the perspectives and experiences of women and HCPs. Although large variation exists in the reported PFMT parameters, PFMT delivered via DTs is promising in terms of improving UI symptoms and QoL. To further optimize UI outcomes and promote long-term adaptation of PFMT, incorporating technological features such as reminders, social media, and gamification, together with other facilitators such as support from HCPs, could be beneficial for women with UI. A greater understanding is required of how women from different cultures and stages in life regard the acceptability, design, development, and effectiveness of PFMT DTs. This is essential to ensure that the quality and content are appropriate and inclusive so that all women and clinicians can have confidence in using these technologies.

## Appendix

Multimedia Appendix 1 Search strategy.

Multimedia Appendix 2 Summary of the included primary and supplementary papers (N=89) and summary of the inclusion and exclusion criteria for the primary and supplementary papers.

Multimedia Appendix 3 Characteristics of the included studies and participants.

Multimedia Appendix 4 Outcomes related to urinary incontinence symptoms and satisfaction with and adherence to the pelvic floor muscle training program delivered via digital technologies.

Multimedia Appendix 5 Summary of main themes and facilitators and barriers from the qualitative studies (N=11).

## Abbreviations

DT: digital technology
GROUP: *Group Rehabilitation Or IndividUal Physiotherapy for Urinary Incontinence in Aging Women*
HCP: health care provider
mHealth: mobile health
PFM: pelvic floor muscle
PFMT: pelvic floor muscle training
PRISMA-ScR: Preferred Reporting Items for Systematic Reviews and Meta-Analyses extension for Scoping Reviews
QoL: quality of life
RCT: randomized controlled trial
UI: urinary incontinence
WHO: World Health Organization
