# Supplementary material for: Digital Technologies for Women’s Pelvic Floor Muscle Training to Manage Urinary Incontinence Across Their Life Course: Scoping Review
Source: JMIR Mhealth Uhealth. 2023 Jul 5;11:e44929. doi: 10.2196/44929 (PMC10357376; doi:10.2196/44929)
Supplement: Multimedia Appendix 1 [file mhealth_v11i1e44929_app1.docx]

**Multimedia Appendix 1. Search Strategy**

**Table S1. Index terms and keywords for searching databases.**

| **Database** | **Keywords** |
| --- | --- |
|  |  |
| AMED, EMBASE and PsychINFO | (Smart phone) OR (Smartphone) OR (Cellphone) OR (Smart device) OR (Smart technology) OR (Internet-based) OR (Web-based) OR (Telerehabilitation) OR (Digital health) OR (Telemedicine) OR (Telehealth) OR (apps) OR ("Mobile application") OR ("mobile health") OR (mHealth) OR ("e-health") OR (eHealth)  AND  (Urine Incontinence) OR (Urinary incontinence) OR (Pelvic floor muscle) OR (Pelvic floor muscle training) OR (PFMT) OR (Kegel exercise) OR (Pelvic floor exercise) OR (postpartum) OR (pregnancy) OR (Levator ani) OR (Menopause) OR ("Genitourinary syndrome")  NOT  (Enuresis) OR (Male) |
| CINAHL | "(Smart phone)” OR (Smartphone) OR (Cellphone) OR (Smart device) OR (Smart technology) OR (Internet-based) OR (Web-based) OR (Telerehabilitation) OR (Digital health) OR (Telemedicine) OR (Telehealth) OR (apps) OR ("Mobile application") OR ("mobile health") OR (mHealth) OR ("e-health") OR (eHealth)  AND  (Urine Incontinence) OR (urinary incontinence) OR (Pelvic floor muscle) OR (Pelvic floor muscle training) OR (PFMT) OR (Kegel exercise) OR (Pelvic floor exercise) OR (Postpartum) OR (Pregnancy) OR (Levator ani) OR (Menopause) OR ("Genitourinary syndrome")  NOT  (Enuresis) OR (Male) |
| MEDLINE | (Smart phone) OR (Smartphone) OR (Cellphone) OR (Smart device) OR (Smart technology) OR (Internet-based) OR (Web-based) OR (Telerehabilitation) OR (Digital health) OR (Telemedicine) OR (Telehealth) OR (apps) OR ("Mobile application") OR ("Mobile health") OR (mHealth) OR ("e-health") OR (eHealth) )  AND  (Urine Incontinence) OR (Urinary incontinence) OR (Pelvic floor muscle) OR (Pelvic floor muscle training) OR (PFMT) OR (Kegel exercise) OR (Pelvic floor exercise) OR (Postpartum) OR (Pregnancy) OR (Levator ani) OR (Menopause) OR ("Genitourinary syndrome")  NOT  (Enuresis) OR (Male) |
| PubMed^a^ | (Smart phone) OR (Smart device)) OR (Smart technology) OR (Technology) OR (Internet-based) OR (Web-based) OR (Telerehabilitation) OR (Digital health) OR (Telemedicine) OR (Telehealth) OR (apps) OR (“Mobile application”) OR (“Mobile health”) OR (Health) OR (“Health”) OR (“e-health”) OR (eHealth)  AND  (Urine Incontinence) OR (urinary incontinence) OR (Pelvic floor muscle) OR (Pelvic floor muscle training) OR (PFMT) OR (Kegel exercise) OR (Pelvic floor exercise) OR (Postpartum) OR (Pregnancy)  NOT  (Enuresis) |
| Scopus | (Urine AND Incontinence) OR (Urinary AND Incontinence) OR (Pelvic AND Floor AND Muscle) OR (Pelvic AND Floor AND Muscle AND Training) OR (pfmt) OR (Kegel AND Exercise) OR (Pelvic AND Floor AND Exercise) OR (Postpartum) OR (Pregnancy) OR (Levator AND Ani) OR (Menopause) OR (“Genitourinary syndrome”)  AND  (Smart AND Phone) OR (Smartphone) OR (Cell AND Phone) OR (Smart AND Device) OR (Smart AND Technology) OR (Internet-based) OR (Web-based) OR (Telerehabilitation) OR (Digital AND Health) OR (Telemedicine) OR (Telehealth) OR (apps) OR (“Mobile application”) OR (“Mobile health”)  AND NOT  (Enuresis) OR (Male) |
| SPORTDiscus | (Urine incontinence) OR (Urinary incontinence) OR (Pelvic floor muscle) OR (Pelvic floor muscle training) OR (PFMT) OR (Kegel exercise) OR (Pelvic floor exercise) OR (Postpartum) OR (Pregnancy) OR (Levator ani) OR (Menopause) OR ("Genitourinary syndrome")  AND  (Smart phone) OR (Smartphone) OR (cellphone) OR (Smart device) OR (Smart technology) OR (Internet-based) OR (Web-based) OR (Telerehabilitation) OR (Digital health) OR (Telemedicine) OR (Telehealth) OR (apps) OR ("Mobile application") OR ("mobile health") OR (mHealth) OR ("e-health") OR (eHealth)  NOT  (Enuresis) OR (Male) |

^a^PubMed was used in preliminary search only.
