# Supplementary material for: Digital Technologies for Women’s Pelvic Floor Muscle Training to Manage Urinary Incontinence Across Their Life Course: Scoping Review
Source: JMIR Mhealth Uhealth. 2023 Jul 5;11:e44929. doi: 10.2196/44929 (PMC10357376; doi:10.2196/44929)
Supplement: Multimedia Appendix 2 [file mhealth_v11i1e44929_app2.docx]

**Multimedia Appendix 2.**

**Table S1. Summary of included primary and supplementary papers (*N*=89).**

| **Primary article** | | | **Supplementary articles linking to primary** | | | |
| --- | --- | --- | --- | --- | --- | --- |
|  | | | | | | |
| ID | Reference | Type | | ID | Reference | Type |
| 1 | Anglès-Acedo et al [37] | Case series, pilot (abstract) | |  |  |  |
| 2 | Anglès-Acedo et al [38] | Qualitative | |  |  |  |
| 3 | Araujo et al [39] | RCT^a^ | | 3a | Juliato et al [89] | Abstract |
| 4 | Asklund et al [5] | RCT | | 4a | Nyström et al [90] | Abstract |
|  |  |  | | 4b | Hoffman et al [91] | 2-year follow up |
|  |  |  | | 4c | Sjöström et al [82] | Secondary analysis, cost-utility |
|  |  |  | | 4d | Joob & Wiwanitkit [92] | Letter to the editor |
|  |  |  | | 4e | Nyström et al [79] | Secondary analysis, success factors |
|  |  |  | | 4f | Nyström et al [93] | Author comments on 4d, 4e |
|  |  |  | | 4g | Asklund et al [78] | Qualitative research |
| 5 | Asklund & Samuelsson [66] | Cross-sectional (abstract) | |  |  |  |
| 6 | Åström et al [55] | Cross-sectional, secondary analysis of 3 RCTs | |  |  |  |
| 7 | Barbato et al [40] | Quasi-experimental | |  |  |  |
| 8 | Bokne et al [41] | Quasi-experimental | |  |  |  |
| 9 | Campbell et al [42] | Mixed methods (quasi-experimental, qualitative), protocol | |  |  |  |
| 10 | Carrión Pérez et al [43] | RCT, pilot | |  |  |  |
| 11 | Coggins et al [44] | Cross-sectional, survey (abstract) | | 11a | Coggins et al [94] | Cross-sectional (abstract) |
| 12 | Conlan et al [45] | Case series | |  |  |  |
| 13 | Cornelius [71] | Cohort (abstract) | | 13a | Starr et al [83] | Case report |
|  |  |  | | 13b | Cornelius et al [95] | Comparison RCT and cohort (abstract) |
|  |  |  | | 13c | Cornelius et al [85] | Cohort (conference presentation) |
| 14 | Dufour et al [67] | Mixed methods (RCT, qualitative), pilot | | 14a | Dufour et al [96] | Abstract |
| 15 | Firet et al [56] | Mixed methods (quasi-experimental, qualitative), protocol | | 15a | Firet et al [31] | Qualitative research |
| 16 | Firet et al [80] | Qualitative, GPs | |  |  |  |
| 17 | Firet et al [46] | Qualitative | |  |  |  |
| 18 | Fischer Blosfeld et al [57] | RCT | | 18a | Latorre et al [11] | App development study |
| 19 | Goode et al [58] | Quasi-experimental, pilot | |  |  |  |
| 20 | Grant & Currie [77] | Qualitative research | |  |  |  |
| 21 | Han et al [72] | Cross-sectional, survey | | 21a | Han et al [97] | Abstract |
| 22 | Hui et al [59] | RCT | |  |  |  |
| 23 | Jaffar et al [60] | RCT, pilot, protocol | | 23a | Sidik et al [81] | RCT, protocol |
| 24 | Kinouchi & Ohashi [68] | Case control | |  |  |  |
| 25 | Li et al [69] | Cross-sectional, survey | |  |  |  |
| 26 | Li et al [47] | Mixed methods (RCT, qualitative), protocol | |  |  |  |
| 27 | Loohuis et al [61] | RCT | | 27a | Loohuis [35] | Note/commentary. Translated from Dutch |
|  |  |  | | 27b | Loohuis et al [12] | Mixed methods (RCT, qualitative), protocol |
|  |  |  | | 27c | Loohuis et al [98] | Abstract |
|  |  |  | | 27d | Heidelbaug [99] | Editorial |
|  |  |  | | 27e | Loohuis et al [100] | 1-year follow up |
|  |  |  | | 27f | Wessels et al [87] | Qualitative research |
| 28 | Moossdorff-Steinhauser et al [52] | RCT, protocol | |  |  |  |
| 29 | Moretti [36] | Case series. Translated from Portuguese | | 29a | Moretti et al [101] | Case series |
|  |  |  | | 29b | Silva et al [102] | Case series |
| 30 | Nyström et al [73] | Cohort, abstract | |  |  |  |
| 31 | Pedofsky et al [48] | Qualitative | | 31a | Cacciari et al [103] | Cohort |
|  |  |  | | 31b | Pedofsky et al [84] | Abstract |
| 32 | Pla et al [49] | Case series, pilot | |  |  |  |
| 33 | Pulliam et al [62] | Quasi-experimental, pilot (abstract) | | 33a | Rosenblatt et al [104] | Abstract |
|  |  |  | | 33b | Rosenblatt et al [105] | Quasi-experimental, pilot |
|  |  |  | | 33c | Bohorquez et al [106] | Case series |
| 34 | Robson [74] | Cross-sectional, survey | |  |  |  |
| 35 | Rygh et al [63] | Cohort | | 35a | Nyström et al [107] | Cohort |
| 36 | Saboia et al [75] | Validation study | |  |  |  |
| 37 | Samuelsson et al [50] | Cohort, abstract | |  |  |  |
| 38 | Shelly [76] | Case report | |  |  |  |
| 39 | Sjöström et al [6] | RCT | | 39a | Sjöström et al [108] | Conference abstract |
|  |  |  | | 39b | Björk et al [86] | Qualitative |
|  |  |  | | 39c | Griebling [109] | Letter to the editor |
|  |  |  | | 39d | Sjöström et al [88] | 1 & 2-year follow up |
|  |  |  | | 39e | Sjöström et al [110] | Secondary analysis, cost-effectiveness |
|  |  |  | | 39f | Lindh et al [111] | Secondary analysis, predictors of success |
|  |  |  | | 39g | Wein [112] | Letter to the editor |
| 40 | Smith [53] | RCT (abstract) | | 40a | Wilson Edwards et al [113] | Abstract |
| 41 | Von Au et al [70] | Cross-sectional | | 41a | Cabral et al [114] | Abstract |
|  |  |  | | 41b | Friedrich et al [115] | Cross-sectional (abstract) |
| 42 | Wadensten et al [64] | RCT | | 42a | Wadensten et al [116] | Abstract, app algorithm development |
|  |  |  | | 42b | Wadensten et al [117] | Abstract |
| 43 | Wang et al [51] | RCT | |  |  |  |
| 44 | Weinstein et al [54] | RCT, protocol | |  |  |  |
| 45 | Wessels et al [65] | Qualitative research | | 45a | Wessels et al [118] | Abstract |

^a^RCT: randomised controlled trial.

**Table S2. Summary of inclusion and exclusion criteria for the primary and supplementary papers.**

| **Inclusion/exclusion criteria** | | | |
| --- | --- | --- | --- |
|  | | | |
| ID | Reference | Inclusion criteria | Exclusion criteria |
| 1 | Anglès-Acedo et al [37] | 18–75 years, mild or moderate SUI | NI^a^ |
| 2 | Anglès-Acedo et al [38] | Stage 1: women, mild or moderate SUI^b^, able to perform PFMT^c^ | NI |
| 3 | Araujo et al [39] | Women, self-reported SUI symptoms (SUI diagnosis based on a demonstration of urinary leakage on straining or coughing), for those presenting MUI^d^ the predominant type was SUI based on the self-reported symptoms, using Questionnaire for Urinary Incontinence Diagnosis (QUID) | Neurologic impairment that affects comprehension, symptoms suggestive of neurogenic bladder (dribbling stream when urinating, inability to fully empty the bladder, straining during urination, loss of bladder control, and difficulty determining when the bladder is full), alterations in PFM contraction (hyperactivity or complete inability to contract) after initial vaginal palpation, previous PFMT, pelvic organ prolapse (greater than stage I by Pelvic Organ Prolapse Quantification),urinary infection symptoms, previous pelvic floor surgery |
| 3a | Juliato et al [89] | Women with SUI | As for Araujo et al [39] |
| 4 | Asklund et al [5] | ³ 18 years, SUI ³ 1 episode/week for the last 6 months (SUI deﬁned as urine leakage upon coughing, sneezing, or physical activity, and no leakage associated with urgency), access to a smartphone and e-mail, Swedish literacy, women with maximum voided volumes ³ 0.3 L | Pregnancy, previous UI^e^ surgery, present or previous malignancy in the lower abdomen, impaired mobility or sensibility in the legs or lower abdomen, severe psychiatric disorders, macroscopic haematuria, irregular bleeding, difﬁculty passing urine |
| 4a | Nyström et al [90] | NI | NI |
| 4b | Hoffman et al [91] | As for Asklund et al [5] | As for Asklund et al [5] |
| 4c | Sjöström et al [88] | As for Asklund et al [5] | As for Asklund et al [5] |
| 4d | Joob [92] | NA^f^ | NA |
| 4e | Nyström et al [79] | As for Asklund et al [5] | As for Asklund et al [5] |
| 4f | Nyström et al [93] | NA | NA |
| 4g | Asklund et al [78] | Women from the RCT app group who had completed the 3-month follow-up | NI |
| 5 | Asklund & Samuelsson [66] | 18–69 years, pregnant or postnatal, had downloaded the app and answered the inclusion questionnaire between the dates of 16 January 2018 and 15 November 2018 | NI |
| 6 | Åström et al [55] | RCT 1: As for Asklund et al [5]  RCT 2: As for Sjöström et al [6]  RCT 3: Wadensten et al [64] | RCT 1: As for Asklund et al [5]  RCT 2: As for Sjöström et al [6]  RCT 3: Wadensten et al [64] |
| 7 | Barbato et al [40] | 21–60 years, self-reported SUI, able to speak and read English fluently, able to access and navigate the internet, able to walk without assistance, move with full range of motion, if using oral contraceptives on a stable dose for at least 3 months | Taking medication to treat SUI, under active treatment for SUI, prior surgery for SUI, pregnant or planning on becoming pregnant, < 6 months postpartum, neurological condition affecting SUI, a patient of one of the co-investigators |
| 8 | Bokne et al [41] | Women actively seeking treatment for SUI |  |
| 9 | Campbell et al [42] | Phase 1: HCPs who women seek help from in the first instance  Phase 2: Female athletes or regular exercisers, ≥ 18 years, self-report symptoms of UI  Phase 3: recruited from phase 2 | Phase 2: women new to sport within the last year, pregnant or < 1 year postnatal, commenced estrogen or anticholinergic treatment within the previous 3 months, ongoing physiotherapy/ continence advice treatment elsewhere within the previous year, existing neurological condition that may contribute to UI, unable to read or understand English |
| 10 | Carrión Pérez et al [43] | Women with SUI, with minimal skills in the use of new technologies | Neurogenic, oncologic, UUI^g^ or MUI, uterine or > 2nd degree bladder and/or rectal prolapse, prior incontinence surgery, speciﬁc pharmacological treatment in the previous 6 months, ongoing genitourinary infection, use a pacemaker |
| 11 | Coggins et al [44] | Elvie device users | NI |
| 11a | Coggins et al [94] | Elvie device users | NI |
| 12 | Conlan et al [45] | Women, 18–75 years, self-reported SUI, living in a community without access to a continence-trained physiotherapist, have access to a computer with internet service able to support video conferencing computer software (SkypeTM) connection or a telephone connection, ability to read and write English | Previous pelvic surgery or radiation, pregnancy, known malignancy in lower abdomen, neurological conditions, severe psychiatric disorders, difficulty passing urine, visible blood in the urine, history of recent UTI^h^ |
| 13 | Cornelius [71] | PeriCoach system users (latest version) | NI |
| 13a | Starr et al [83] | SUI, access to a smartphone or tablet | NI |
| 13b | Cornelius et al [95] | NI | NI |
| 13c | Cornelius et al [85] | PeriCoach V3 users | NI |
| 14 | Dufour et al [67] | Women who went on to have a vaginal birth or caesarean section, at a timepoint within 21 days of delivery | Unable to understand and read English, directed by their caregivers to not insert anything into their vagina |
| 14a | Dufour et al [96] | NI | NI |
| 15 | Firet et al [56] | Women aged > 18 years old, reported SUI (MUI also included, these participants informed intervention is specifically designed for SUI), capable of understanding Dutch language, internet access | Participation in another therapy program or trial for SUI, surgery for UI in the last 6 months, PFMT from a pelvic physiotherapist in the last 6 months, pregnancy, vaginal delivery in the last 6 months, neurological disease affecting lower limbs (e.g., Parkinsons disease, multiple sclerosis, cerebrovascular incident), malignancy in lower abdomen currently or in the past 5 years (colon, uterus, cervix, bladder, ovary, or vagina) |
| 15a | Firet et al [31] | As for Firet et al [56] | As for Firet et al [56] |
| 16 | Firet et al [80] | Purposive sampling of GPs recruited by 10 other GPs of the Dutch College of General Practitioners expert group on urogynaecological diseases, whose practice locations were distributed throughout the Netherlands. Each GP from this expert group asked to approach two GPs from their region who did not belong to the urogynaecology expertise group | NI |
| 17 | Firet et al [46] | Women, ≥ 18 years, experiencing predominantly SUI, history of asking for and receiving help regarding SUI at least once, no history of serious illness, able to communicate in Dutch | NI |
| 18 | Fischer Blosfield et al [57] | Women with SUI, UUI, MUI, 18-59 years, with objective demonstration of urinary loss | Pregnant, up to 6 months postpartum, with prolapse ≥ Stage 3 according to the Pelvic Organ Prolapse-Quantification, with urinary infection, intrapelvic tumours, pelvic pain preventing performance of the available therapies, pelvic surgery < 6 months, carrier of pacemaker or intrauterine device, missed physical therapy twice in a row |
| 18a | Latorre et al [11] | NA | NA |
| 19 | Goode et al [58] | Women with SUI, UUI, or MUI, ≥ 2 times per week for 3 or more months, if taking medication for overactive bladder dosage stable for at least 1 month or the drug discontinued for at least 1 month | Prior behavioural treatment for UI, without internet access |
| 20 | Grant & Currie [77] | ≥ 18 years, given birth to ≥ 1 children in the last 5 years | NI |
| 21 | Han et al [72] | Patients: women, > 18 years, had smart phones, with interest in downloading and using the mobile app Bwom  Providers: gynecologists, urogynecologists, pelvic floor physical therapy specialists, women’s health nurse practitioners, or midwives | Significant physical or medical conditions that would prevent them doing basic exercises such as Kegels, leg lifts, sit ups, and kneeling, scheduling conflicts preventing using the mobile application for 2 weeks |
| 21a | Han et al [97] | English-speaking women ≥ 18 years interested in PFMT, with or without UI or prolapse symptoms | Significant physical or medical conditions that would prevent them from doing PFMT |
| 22 | Hui et al [59] | Women, ≥ 60 years, SUI or UUI symptoms, ≥ 1 incontinence episode in a week | Active UTI, post-void residual volume by ultrasound of > 150ml, 3rd degree uterine prolapse, already receiving treatment for urinary symptoms |
| 23 | Jaffar et al [60] | Pregnant women, > 18 years, any parity at 26–27 weeks’ gestation, with SUI or MUI according to the ICIQ-UI-SF^j^, Malaysian citizens | Pregnant with chronic medical problem(s) before pregnancy, complicated pregnancies, or conditions with which it is not advisable to practise PFMT, non-Malaysian citizens |
| 23a | Sidik et al [81] | Women, ≥ 18 years, primipara and multiparas at 26–27 weeks’ gestation with UI scores of 3–18 according to the ICIQ-UI-SF, Malaysian citizens with Malay literacy (who consist of Malay, Chinese, Indian and native ethnicities) | Pregnancy with a chronic medical problem(s) before pregnancy (diabetes, hypertension, human immunodeficiency virus positive, a neurological condition affecting bladder control, stroke, pelvic organ prolapse), complicated pregnancies or conditions which are not advisable to practice PFMT (such as pre-eclampsia, persistent bleeding, preterm, labour, incompetent cervix, acute febrile infection, foetal growth restriction or placenta previa and cephalopelvic  disproportion) |
| 24 | Kinouchi & Ohashi 2018 [68] | Intervention: Women, > 1 month postpartum, undergone vaginal delivery, have a smartphone  Control: Postpartum who had vaginal delivery | History of pelvic surgery and cerebral infarction, current hypertension, diabetes, haemorrhage, cystitis, neurological disease of the urinary system, chronic cough, diuretic use |
| 25 | Li et al 2020 [69] | Women, ≥ 18 years, gave birth after 37 weeks gestation, immediately postpartum (at routine six weeks of postpartum control) | NI |
| 26 | Li et al [47] | Women, ≥ 18 years, have a singleton pregnancy according to ultrasonographic evaluation, at 24–28 gestational weeks, access to a mobile phone and the internet, SUI symptoms with ≥ 1 leakage episodes over the past 4 weeks (SUI symptoms are assessed by asking the question “When does urine leak?”) | Psychiatric illness or cognitive impairment, previous UI, pelvic organ prolapse or pelvic surgery, serious comorbidities or complications like heart disease, diabetes mellitus, hypertensive disorder of pregnancy, threatened abortion, placenta previa, placental abruption and premature rupture of membranes, foetal growth restriction, and amniotic fluid abnormalities |
| 27 | Loohuis et al [61] | Women, ≥ 18 years, self-reported SUI, UUI, MUI, ≥ 2 episodes of UI per week according to the Three Incontinence Questions (3IQ) questionnaire, access to smartphone or tablet, wished to be treated | Unable to complete questionnaire in Dutch, indwelling urinary catheter, urogenital malignancy, previous surgery for UI, treatment for UI in the previous year (pharmacological or non-pharmacological), terminal or serious illness, cognitive impairment, psychiatric illness, UTI (dipstick, and if negative, dipslide or urine culture), overflow or continuous UI, pregnancy, or recent childbirth (< 6 months ago) |
| 27a | Loohuis [35] | NA | NA |
| 27b | Loohuis et al [12] | As for Loohuis et al [61] | As for Loohuis et al [61] |
| 27c | Loohuis et al [98] | As for Loohuis et al [61] | As for Loohuis et al [61] |
| 27d | Heidelbaugh [99] | NA | NA |
| 27e | Loohuis et al [100] | As for Loohuis et al [61] | As for Loohuis et al [61] |
| 27f | Wessels et al [87] | Purposive sampling of women who had completed the 12-month follow-up from the RCT | As for Loohuis et al [61] |
| 28 | Moossdorff- Steinhauser et al [52] | Women, ≥ 18 years, UI (stress or mixed with predominant stress), > 3 score on the ICIQ-UI-SF,  motivated for participation in the Motherfit programme, speak and understand the Dutch language, ability to read and fill in forms independently, have a mobile app (mApp) on a tablet (Apple or Android) available | UI prior to first pregnancy, UI still existing during pregnancy, high-risk pregnancy resulting in a contra-indication for performing intensive pelvic-floor-muscle exercises (e.g., placenta praevia, vaginal blood loss, preterm uterine contractions), suffering from significant exercise limitations or co-morbidities (physical or psychological) that would restrain a participation in Motherfit group therapy, history of chronic neurological disorders or diseases related to UI (e.g. multiple sclerosis, cerebrovascular accident, diabetes mellitus (during ≥ 1 year with glycated hemoglobin (HbA1c) > 10 mmol/l)) UTI (urine-sediment, urine culture), history of anti-incontinence or urogynaecological surgery, those expected to be lost to follow-up (e.g. because of a planned change of residency), recent pelvic physiotherapy (< 6 months), refusal to use a mApp |
| 29 | Moretti [36] | Phase 1: women, 18–35 years, eutrophic (BMI between 18.5–24.99), nulliparous, in menacme  Phase 4: same inclusion/exclusion criteria, but no restriction on BMI and parity | History of urogynaecological and neuromuscular diseases, chronic constipation, diabetes, connective tissue diseases, anal fissure, active haemorrhoids, cognitive deficit |
| 29a | Moretti et al [101] | As for Phase 1, Moretti [36] | As for Moretti [36] |
| 29b | Silva 2019 [102] | NA | NA |
| 30 | Nyström et al [73] | Participants who downloaded the app between May 2015 and April 2017, ≥ 18 years, with UI (defined as reporting any frequency and any amount of leakage in the ICIQ-UI-SF questionnaire) | NI |
| 31 | Pedofsky et al [48] | Women, > 18 years, comfortable using an intravaginal device, own a smartphone | Those familiar with the project (e.g., part of the research team, taken part in a previous usability or research trial, before focus group 1). Pregnant, menstruating, had vaginal infections or bulging pelvic organ prolapse |
| 31a | Cacciari et al [103] | NA | NA |
| 31b | Pedofsky et al [84] | Women with some experience of UI | NI |
| 32 | Pla et al [49] | NI | NI |
| 33 | Pulliam et al [62] | NI | NI |
| 33a | Rosenblatt et al [104] | Pre- and perimenopausal women with mild-moderate SUI or MUI | NI |
| 33b | Rosenblatt et al [105] | Women who indicated SUI or stress‐dominant MUI using the Medical, Epidemiologic, and Social Aspects of Aging questionnaire, ≥ 18‐years old, premenopausal | History of lower back or pelvic surgery; pregnant or < 12‐months postpartum; > 3 vaginal deliveries or any prior operative delivery; self‐reported pelvic organ prolapse symptoms; history of supervised PFMT within 12 months; currently taking medication for UI |
| 33c | Bohorquez et al [106] | NA | NA |
| 34 | Robson [74] | App users who responded to survey between June to September 2015 | NI |
| 35 | Rygh et al [63] | Tät app users, identified as female, 18–99 years, not pregnant or given birth within the last 3 months, stated they downloaded the app to improve their incontinence or train preventively  Follow-up: those who responded to questionnaire within 89–135 days, were incontinent at baseline | Were researchers/ health-care providers (as stated on app), just curious about the app  Follow-up: pregnant or given birth within the last 3 months |
| 35a | Nyström et al [107] | Those who responded to the PGI-I within 89–135 days, self-stated female sex, ≥ 18 years, UI defined according to the ICIQ-UI-SF | Currently pregnant or postpartum (< 3 months) at baseline or follow-up |
| 36 | Saboia et al [75] | Women, ≥ 18 years, postpartum in the immediate post-delivery period, had given birth to a full-term new-born vaginally, had a cell phone or similar device compatible with the Continence App | NI |
| 37 | Samuelsson et al [50] | Women, who downloaded app and filled out associated questionnaire between May-November 2015, and follow-up questionnaire from August 2015-February 2016 | NI |
| 38 | Shelly [76] | NI | NI |
| 39 | Sjöström et al [6] | 18–70 years, SUI ≥ 1 time per week (determined from ICIQ-UI SF, 2-day bladder diary and telephone interview with urotherapist), access to computer with internet connection | Pregnancy, previous UI surgery, known malignancy in lower abdomen, difficulties with passing urine, macroscopic haematuria, intermenstrual bleedings, severe psychiatric disorders, or Hospital Anxiety and Depression Scale (HADS) score > 15 for depression or anxiety, neurological disease with affection on sensibility in legs or lower abdomen |
| 39a | Sjöström et al [108] | As for Sjöström et al [6] | As for Sjöström et al [6] |
| 39b | Björk et al [86] | Select sample of women from the RCT: 18–70 years, SUI ≥ once weekly | NI |
| 39c | Griebling [109] | NA | NA |
| 39d | Sjöström et al [88] | As for Sjöström et al [6] | As for Sjöström et al [6] |
| 39e | Sjöström et al [110] | As for Sjöström et al [6] | As for Sjöström et al [6] |
| 39f | Lindh et al [111] | As for Sjöström et al [6] | As for Sjöström et al [6] |
| 39g | Wein [112] | NA | NA |
| 40 | Smith [53] | Women, ≥ 18 years, who met the inclusion criteria | NI |
| 40a | Wilson Edwards et al [113] | Women, ≥ 18 years, who met the inclusion criteria | NI |
| 41 | von Au et al [70] | Women, ≥ 18 years, used Pelvina app (enrolled via app usage), consented to data evaluation during app onboarding process, had complete datasets | NI |
| 41a | Cabral et al [114] | NI | NI |
| 41b | Friedrich et al [115] | NI | NI |
| 42 | Wadensten et al [64] | Women ≥ 18 years, experiencing UUI or MUI with ≥ 2 leakages/week and symptoms lasting for ≥ 12 months, access to a smartphone (at least iOS 8.0 or Android 4.0.3), ability to send and receive email | Pregnancy, using another PFMT app, use mirabegron or antimuscarinic drugs, incontinence surgery within the last 5 years, red-flag symptoms and certain medical conditions (e.g., painful urgency, previous pyelonephritis, ≥ 3 UTIs in the last 12 months, dysuria, visible haematuria, non-investigated bladder-emptying difficulties, metrorrhagia, cancer in the pelvic area, bladder, or bowels, decreased mobility or sensitivity in the legs or pelvic area, history of stroke, neurological disease, diabetes), respondents with a max. voided volume of ≤ 150 mL |
| 42a | Wadensten et al [116] | As for Wadensten et al [64] | As for Wadensten et al [64] |
| 42b | Wadensten et al [117] | As for Wadensten et al [64] | As for Wadensten et al [64] |
| 43 | Wang et al [51] | Women, 20-34 years, nulliparous with a singleton pregnancy and cephalic presentation at 30–32 gestational age, SUI symptoms with episode frequency ≥ 1 per month during the last 3 months (SUI defined as urine leakage on coughing, sneezing, laughing or physical activities), being continent before pregnancy, understand the study procedure, willing to participate in the study | Severe comorbidities like placenta previa, threatened premature labour or pregnancy-induced hypertension, history of chronic cough, constipation, pelvic surgery, spinal surgery, urinary system disease (e.g., active urinary tract infection) or diabetes mellitus, indications of caesarean section or contraindications of vaginal birth |
| 44 | Weinstein et al [54] | Women, ≥ 18 years old, capable of giving informed consent, possess a compatible smartphone, self-reported SUI/SMUI symptoms for at ≥ 3 months, SUI predominant mixed incontinence or SUI only based on MESA stress symptom score, English speaking, postmenopausal, post hysterectomy or willing to use an acceptable method of birth control for the duration of the study, able to complete a bladder diary using the ClaimIT!2020 app, able to complete electronic surveys and upload data, willing to provide contact information and respond to communication from the study team, willing to participate in the 8-week study with follow-up at 6- and 12- months, refrain from other treatments for SUI/SMUI for the first 8 weeks | Self-reported absence of vagina, report seeing or feeling a vaginal bulge, neuromuscular disease that may contribute to UI (e.g., Parkinson’s disease, dementia, stroke), non-ambulatory, pregnant or < 6 months postpartum, currently (or within last month) breast-feeding, prior surgery for SUI, previous PFMT: 2 visits within the last 3 months under supervised therapeutic plan of care, taking or has taken within the last 2 months medications to treat UI, prior augmentation cystoplasty or artificial sphincter, implanted nerves stimulator for urinary symptoms active within the past 60 days, history of impaired cognitive function, contraindication to the use of a vaginal probe, inability to understand instructions for device use, unable to suitably operate the smartphone app |
| 45 | Wessels et al [65] | ≥ 18 years, self-reported UI at least twice a week, wanted treatment, had access to a smartphone or tablet | Indwelling urinary catheter, urogenital malignancy, previous UI surgery, treatment for UI in the previous year, terminal or serious illness, cognitive impairment, psychiatric illness (reported by their GP), overflow or continuous UI, pregnancy or recent childbirth (< 6 months ago), inability to complete questionnaire in Dutch |
| 45a | Wessels et al [118] | As for Wessels et al [65] | As for Wessels et al [65] |

^a^NI: not indicated.

^b^SUI: stress urinary incontinence.

^c^PFMT: pelvic floor muscle training.

^d^MUI: mixed urinary incontinence.

^e^UI: urinary incontinence.

^f^NA: not applicable.

^g^UUI: urge urinary incontinence.

^h^UTI: urinary tract infection.

^i^ICS-POPQ: International Continence Society-Pelvic Organ Prolapse Quantification.

^j^ICIQ-UI-SF: International Consultation on Incontinence Questionnaire-Urinary Incontinence-Short Form.
