# Supplementary material for: Digital Technologies for Women’s Pelvic Floor Muscle Training to Manage Urinary Incontinence Across Their Life Course: Scoping Review
Source: JMIR Mhealth Uhealth. 2023 Jul 5;11:e44929. doi: 10.2196/44929 (PMC10357376; doi:10.2196/44929)
Supplement: Multimedia Appendix 3 [file mhealth_v11i1e44929_app3.docx]

**Multimedia Appendix 3.**

**Table S1. Characteristics of included studies and participants.**

| **Study characteristics** | | | | | | | **Participant characteristics** | | | | |
| --- | --- | --- | --- | --- | --- | --- | --- | --- | --- | --- | --- |
| **ID** | **Reference** | **Country of origin** | **Design** | **Aims** | **Quality rating** | **Sample size, n** | **Age, years, mean (SD)** | **Stage of life** | **Level of education, n (%)** | **UI^a^ type** | **Duration of UI symptoms, months, mean (SD)** |
|  |  |  |  |  |  |  |  |  |  |  |  |
| 1 | Anglès-Acedo et al [37] | Spain | Case series, pilot (abstract) | Functionality | Poor | 21 | 45 (32–67)^b^ | NI | NI^c^ | Stress | NI |
| 2 | Anglès-Acedo et al [38] | The Netherlands, Spain, Finland | Qualitative | Explore women’s perspectives | High | Stage 1: 22  Stage 3: 9^d^ | Stage 1: 47 (29–71)^e^  Stage 3: 41 (25–61)^e^ | NI | NI | Stress | 59.2 (151.7) |
| 3 | Araujo et al [39] | Brazil | RCT | Adherence | Fair | I^f^: 17  C^g^: 16 | I: 47.2 (10.6)  C: 53.3 (13.3) | NI | NI | Stress | NI |
| 4 | Asklund et al [5] | Sweden | RCT | Effectiveness | High | I: 62  C: 61 | Total: 44.7  I: 44.8 (9.7)  C: 44.7 (9.1) | NI | ≥ 3 years university  I: 52 (83.9)  C: 46 (75.4) | Stress | ≥ 6 months |
| 4b | Hoffman et al [91] | Sweden | RCT, 2-year follow up | Effectiveness | High | I: 46  C: 0 | Total: 44.2 (10.3) | NI | ≥ 3 years university  37 (80.4) | Stress | ≥ 6 months |
| 4g | Asklund et al [78] | Sweden | Qualitative | Explore women’s experiences | High | 15 | 47 (27-72)^e^ | NI | NI | Stress | NI |
| 5 | Asklund & Samuelsson [66] | Sweden | Cross-sectional, survey | Explore app use and user characteristics | Fair | 10,588 | Total: 31 (4.9)  Pregnant: 31 (5.4)  Postpartum: 31 (4.6) | Pregnancy, postpartum | University education  Total: 7704 (73)  Pregnant: 3477 (74)  Postpartum: 4227 (72) | Healthy, incontinent^h^ | NI |
| 6 | Åström [55] | Sweden | Cross-sectional, secondary analysis of 3 RCTs | Impact of UI on quality of life | – | 496 | 50.1 (11.0) | NI | > 3 years university  Total: 312 (62.9)  RCT 1: 135 (54.0)  RCT 2: 98 (79.7)  RCT 3: 79 (64.2) | Stress, urge, mixed | NI |
| 7 | Barbato et al [40] | USA | Quasi-experimen-tal | Feasibility,  efficacy | Poor | 34 | 45.5 (27–61)^e^ | NI | Degree in higher education: (80) | Stress | 6.6 (3-40)^c^ |
| 8 | Bokne et al [41] | Sweden | Quasi-experimen-tal | Effectiveness,  user characteristics | Fair | I1: 109  I2: 166 | I1: 59.4  I2: 54.5 | NI | Post-secondary education:  I1: 64 (59)  I2: 109 (67) | Stress | NI |
| 9 | Campbell et al [42] | UK | Mixed methods, protocol | RCT feasibility | – | Phase 1: 6-8 local HCPs^j^  Phase 2: 15-20  Phase 3: 6-8 | – | Other - athletic women | – | Stress | – |
| 10 | Carrión Pérez et al [43] | Spain | RCT, pilot | Effectiveness | Fair | I: 10  C: 9 | I: 49 (47–56)^k^  C: 46 (46–49.75)^k^ | NI | Primary  I: 3 (30)  C: 2 (22.2)  ≥ Secondary  I: 7 (70)  C: 7 (77.8) | Stress | ‘Progression time’  I: 36 (18–102)^k^  C: 108 (66–195)^k^ |
| 11 | Coggins et al [44] | UK | Cross-sectional,  survey | User demographics, effectiveness | Poor | 417 | 39 (33–49)^k^ | NI | Not specified | Stress | NI |
| 12 | Conlan et al [45] | Australia | Case series | Efficacy | High | 6 | 36.5 (24–56)^b^ | Pre-menopause,  lactating,  Post-menopause | Year 12: 2 (33.3)  University: 2 (33.3)  Postgraduate: 2 (33.3) | Stress | NI |
| 13 | Cornelius [71] | Australia | Cohort | Assess technique | Poor | 28 | NI | NI | NI | NI | NI |
| 14 | Dufour et al [67] | Canada | Mixed methods, pilot | Acceptability, feasibility, effectiveness | High | I: 13  C: 10 | I: 31 (2.7)  Unknown: n=3  C: 34 (2.2)  Unknown: n=4 | Postpartum | NI | Healthy, incontinent^h^ | NI |
| 15 | Firet et al [56] | The Netherlands | Mixed methods, protocol | Barriers and facilitators, effectiveness | High | 800^l^ | – | NI | – | Stress, mixed | – |
| 15a | Firet et al [31] | The Netherlands | Qualitative | Barriers and facilitators to eHealth interventions for stress UI | High | 20 | mean: 51 | NI | Primary- upper secondary: 3 (15); Post-secondary non-tertiary- Bachelor’s: 5 (25); Master’s: – Doctoral: 12 (60) | Stress | NI |
| 16 | Firet et al [80] | The Netherlands | Qualitative | Explore GPs attitudes toward eHealth interventions for stress UI | High | 13 GPs (9 female) | ≤ 45: 8^m^ > 45: 5^m^ | Other – qualified GPs | – | – | – |
| 17 | Firet et al [46] | The Netherlands | Qualitative | Expectations regarding eHealth intervention | High | 13 | 40-59: 6^m^  60-79: 4^m^  ≥80: 3^m^ | Postpartum /menopause, post-menopause | Primary: 1 (8) Level 1–4: 11 (84)  Level 5–7: 1 (8) | Stress | ≤2 years: 3^m^  2-5 years: 3^m^  ≥5 years: 7^m^ |
| 18 | Fischer Blosfield et al [57] | Brazil | RCT | Effectiveness | Fair | I1: 19  I2: 22  I3: 19  I4: 17 | I1: 46.73 (9.0)  I2: 51.4 (6.6)  I3: 45.84 (6.3)  I4: 48.82 (9.4) | NI | Schooling 1-5 years: I1: 1 (5); I2: 5 (27)  I3: 0; I4: 0  6-10 years: I1: 10 (52); I2: 12 (54); I3: 4 (21); I4: 7 (41)  >10 years: I1: 8 (42); I2: 5 (22); I3: 15 (78); I4: 10 (58) | Stress, urge, mixed | NI |
| 19 | Goode et al [58] | USA | Quasi-experimental, pilot | mHealth development, efficacy | Fair | 29^n^ | 54 (10.4) | Menopause, post-menopause | High school/ GED equivalent: 13 (45); College degree: 16 (55) | Stress, urge, mixed | NI |
| 20 | Grant & Currie [77] | UK | Qualitative research | Perceptions, barriers and facilitators, acceptability of postpartum physical activity and PFMT | High | 31 | Range 28–43 | Postpartum (given birth in last 5 years) | Majority educated to degree or master’s level | – | NI |
| 21 | Han et al^o^ [72] | USA | Cross-sectional, survey | Understandability, actionability | Poor | 25 | 20-30: 24%  31-40: 68%  41-50: 9% | NI | Attended college: 2 (6); Bachelor’s: 10 (29); Master’s: 20 (59); Doctorate: 2 (6) | NI | NI |
| 22 | Hui et al [59] | Hong Kong, China^q^ | RCT | Effectiveness, efficacy | Fair | I: 27  C: 31 | Total: 74 (5)  I: 73.6 (5.5)  C: 73.5 (3.8) | Post-menopause | No education  I: 8 (29.6)  C: 9 (29.0)  Primary  I: 15 (55.6)  C: 19 (61.3)  Secondary  I: 4 (14.8)  C: 3 (9.7) | Stress, urge | NI |
| 23 | Jaffar et al [60] | Malaysia | RCT, pilot, protocol | Feasibility, effectiveness | – | 64 | – | Pregnancy | – | Stress, mixed | – |
| 24 | Kinouchi & Ohashi^r^ [68] | Japan | Case control | Adherence, effectiveness | Fair | I: 31/29  C: 121/29 | I: 34 (31-36)/34 (31–36)^k^  C: 33 (30-36)34 (32–37)^k^ | Postpartum | NI | Healthy, incontinent^h^ | NI |
| 25 | Li et al [69] | China | Cross-sectional, survey | Factors influencing mHealth treatment participation | Fair | 1982^s^ | < 25: 127^m^  25–34: 1354^m^  35–44: 420^m^  > 45: 39^m^  Unknown: 42^m^ | Postpartum | ≤ Middle school 82 (4.1); High school 180 (9.1); > Bachelor’s 1685 (85.0); Unknown: 35 (1.8) | Healthy, stress, urge, mixed^h^ | NI |
| 26 | Li et al [47] | China | Mixed method, protocol | Effectiveness | – | I: 168  C: 168 | – | Pregnancy | – | Stress | – |
| 27 | Loohuis et al^t^ [61] | The Netherlands | RCT | Effectiveness | High | I: 102  C: 93 | Total: 53  I: 53.2 (12.8)  C: 51.3 (10.3) | NI | >High school  I: 53 (52.0)  C: 48 (51.6) | Stress, urge, mixed | I: 7 years (4–14)^g^  C: 8 years (4–13)^g^ |
| 27e | Loohuis et al [100] | The Netherlands | RCT, 1-year follow up | Effectiveness | High | I: 89  C: 83 | Total 54 (23-86)^e^  I: 54.9 (12.2)^e^  C: 52.0 (9.8)^e^ | NI | Higher education level  I: 43 (51.8)  C: 40 (50.6) | Stress, urge, mixed | NI |
| 27f | Wessels et al [87] | The Netherlands | Qualitative | Factors influencing app treatment for UI, barriers and facilitators | High | 17 | Range 35-78 | NI | Primary or secondary: 8 (47.1) | Stress, urge, mixed | 3 months–20 years |
| 28 | Moossdorff-Steinhauser et al [52] | The Netherlands | RCT, protocol  (Motherfit 1) | Efficacy, cost-effectiveness | – | 150^l^ | – | Pregnancy | – | Stress, mixed^p^ | – |
|  |  |  | RCT, protocol  (Motherfit 2) | Efficacy, cost- effectiveness | – | 90^l^ | – | Postpartum | n/a | Stress, mixed^p^ | – |
| 29 | Moretti^u^ [36] | Brazil | Case series | App usability, system satisfaction, device validation | Poor | 15  30 | 24.7 (2.4)  25.8 (3.0) | NI | NI | – | – |
| 30 | Nyström et al^v^ [73] | Sweden | Cohort | Factors associated with self-management, improvement | Fair | 13257 | 40.2 (18–98)^e^ | NI | NI | NI | NI |
| 31 | Pedofsky et al [48] | New Zealand | Qualitative | Use codesign to develop mobile app for pelvic floor muscle training | High | 26 | Range 22–62 | NI | NI | Stress | NI |
| 32 | Pla et al [49] | Spain | Case series, pilot | Evaluate use in managing stress UI | Poor | 7 | 35.9 (11.8) | NI | NI | Stress | NI |
| 33 | Pulliam et al [62] | USA | Quasi-experimental, pilot | Effectiveness, satisfaction | Fair | 23 | 42 (10.7) | Pre-and peri-menopause | NI | Stress, mixed | NI |
| 34 | Robson [74] | UK | Cross-sectional, survey | App user satisfaction | Poor | 464 | NI | NI | NI | NI | NI |
| 35 | Rygh et al^w^ [63] | Sweden | Cohort | User characteristics, effectiveness | Fair | 2672 | 44.4 (13.5) | NI | > 6 years of school 28 (1.0); 7–9 years: 48 (1.8); 10–12 years: 586 (21.9); University: 2010 (75.2) | Stress, urge, mixed | NI |
| 35a | Nyström et al [107] | Sweden | Cohort | User improvement factors | High | 2153 | < 30: 171^k^  30–39: 672^k^  40–49: 567^k^  ≥ 50: 743^k^ | NI | ≤ 9 years of school: 57 (2.6)  10–12 years: 462 (21.5); University or college: 1634 (75.9) | Stress, urge, mixed | NI |
| 36 | Saboia et al^x^ [75] | Brazil | Validation study | App construction and validation to promote adherence | – | 22 | 27 (5.4) | Postpartum | 12 (2.9)^y^ | NI | – |
| 37 | Samuelsson et al^z^ [50] | Sweden | Cohort | User characteristics, efficacy | Fair | 273 | 36.7 (10.1) | NI | Engaged in university studies: 195 (71.4) | Stress | NI |
| 38 | Shelly [76] | USA | Case report | Report case for retraining the pelvic floor muscles | High | 1 | 66 | Parous | NI | Urge | NI |
| 39 | Sjöström et al [6] | Sweden | RCT | Effectiveness | High | I1: 124  I2: 126 | I1: 47.9 (10.6)  I2: 49.4 (9.8) | NI | < 3 years university  I1: 25 (20.2)  I2: 28 (22.2)  ≥ 3 years university  I1: 63 (50.8)  I2: 72 (57.1) | Stress | NI |
| 39d | Sjöström et al [88] | Sweden | RCT 1 & 2-year follow up | Effectiveness |  | I1: 124  I2: 126 | I1: 47.9 (10.6)  I2: 49.4 (9.8) | NI | Primary and lower secondary  I1: 4 (3.2)  I2: 1 (0.8)  Upper secondary  I1: 32 (25.8)  I2: 25 (19.8)  Post-secondary  I1: 88 (71.0)  I2: 100 (79.4) | Stress | NI |
| 39b | Björk et al [86] | Sweden | Qualitative research | Explore women’s experiences using internet-based SUI treatment | High | 21 | 47.6 (30–69)^e^ | NI | NI | Stress | NI |
| 40 | Smith 2017^aa^ [53] | Australia | RCT | Efficacy, satisfaction | Poor | 51 | NI | NI | NI | Stress, mixed^p^ | NI |
| 41 | von Au et al [70] | Germany | Cross-sectional | Data reproducibility,  Patient knowledge | Fair | 293 | 36 (32–44)^k^ | NI | NI | Healthy, incontinent^h^ | NI |
| 42 | Wadensten et al [64] | Sweden | RCT | Efficacy | High | I: 60  C: 63 | Total: 58.3 (9.6)  I: 58.9 (9.2)  C: 57.7 (9.9) | NI | ≥ 3 years university  I: 44 (73.3)  C: 35 (55.6) | Urge, mixed | 1 to 5 years^m^  I: 23  C: 25  > 5 years^m^  I: 37  C: 38 |
| 43 | Wang et al [51] | China | RCT | Effectiveness, Adherence | High | I: 54  C: 54 | Total: 29.1 (2.8)  I: 29.2 (2.6)  C: 29.1 (2.9) | Pregnancy | < Junior college  I: 11 (20.4)  C: 14 (25.9)  Bachelor’s degree  I: 29 (53.7)  C: 28 (51.9)  ≥ Master’s degree  I: 14 (25.9)  C: 12 (22.2) | Stress | Developed during pregnancy |
| 44 | Weinstein et al [54] | USA | RCT, protocol | Efficacy | – | 350^l^ | – | NI | – | Stress, mixed^p^ | – |
| 45 | Wessels et al [65] | The Netherlands | Qualitative research | Experiences, preferences of mobile app for UI treatment | High | 9 | Range 32–68 years | NI | Pre-university education: 3 (33.3)  Secondary vocational education: 1 (11.1)  Senior secondary vocational education: 2 (22.2)  Bachelor’s: 2 (22.2)  Masters: 1 (11.1) | Stress, urge, mixed | Range 18–312 |

^a^UI: urinary incontinence.

^b^reported as median (range).

^c^NI: not indicated.

^d^7 of 9 participants were healthy volunteers and 2 had SUI.

^e^reported as mean (range).

^f^I: intervention.

^g^C: control.

^h^study included participants with and without UI symptoms.

^i^reports quality of life data from participants involved in three previous RCTs ( [5, 6, 64]).

^j^health care professionals (HCPs) recruited for semi-structured interviews as part of phase 1, with phase 2 and 3 recruiting treatment participants and phase 3 consisting of semi-structured interviews.

^k^reported as median (interquartile range).

^l^approximate total number of participants required for the study as identified by the authors.

^m^reported as number of participants.

^n^30% of participants were stated as using overactive bowel medication to maintain improvement in MUI or UUI symptoms as part of prior first in-line treatment

^o^data shown for patient participants only, excluding providers.

^p^mixed UI included if stress symptoms predominant.

^q^not clear: authors of the study based in Hong Kong, two teleconferencing sites based in Norway and Community Centre for seniors based in California USA,

^r^authors conducted propensity score matching with historical controls to allow analysis of data as a quasi-randomised control trial.

^s^739 of 1982 enrolled in study had complaints and symptoms of UI.

^t^sample size provided for intention to treat analysis; per protocol provided in original paper as appendix analysis.

^u^data from thesis provided for Phase 4 trial of device and virtual game app with data from Phase 1 device validation provided in Moretti et al [134].

^v^data shown for app users who responded to baseline questionnaire, of which 1610 provided follow-up data.

^w^data shown for subgroup of app users who responded at 3-month follow up only, of a total 24602 users who responded to baseline questionnaire.

^x^data shown for target group participants recruited as part of the study during app validation stage only, excluding 22 experts also recruited during this stage.

^y^reported as years of study, mean (standard deviation).

^z^data shown for subgroup of app users who responded at 3-month follow up only, of a total 2006 users who responded to baseline questionnaire.
^aa^authors report results available of 51 participants of a total 100 to be included as part of study.
