# Supplementary material for: Digital Technologies for Women’s Pelvic Floor Muscle Training to Manage Urinary Incontinence Across Their Life Course: Scoping Review
Source: JMIR Mhealth Uhealth. 2023 Jul 5;11:e44929. doi: 10.2196/44929 (PMC10357376; doi:10.2196/44929)
Supplement: Multimedia Appendix 4 [file mhealth_v11i1e44929_app4.docx]

**Multimedia Appendix 4.**

**Table S1. Outcomes related to urinary incontinence symptoms.**

| **Article** | | **Study** | **UI symptoms** | |
| --- | --- | --- | --- | --- |
| **ID** | **Author^a^** | **Groups** | **Measures** | **Results** |
|  |  |  |  |  |
| 3 | Araujo et al [39] | I: Mobile app – Diário Saúde  C: Printed PFMT^b^ instructions | ICIQ-VS^c^  ICIQ-UI-SF^d^  QUID^e^ | Between groups: 91% of participants in both groups reported symptom improvement after PFMT. No significant difference between groups in all other outcome measures.  Within groups: significant improvement in both groups for ICIQ-VS (vaginal symptoms, *P* < 0.001; QoL *P* = 0.003); ICIQ-UI-SF (Stress UI symptoms, *P* < 0.001); QUID (Total UI symptoms, *P* < 0.001). |
| 4 | Asklund et al [5] | I: Mobile app – Tät  C: No intervention | ICIQ-UI-SF  ICIQ-LUTSqol^f^  PGI-I^g^  2-day bladder diary | At 3 months: Between groups: significant difference in all outcome measures, favouring I^h^.  ICIQ-UI-SF, I: 7.0 (3.5); C^i^: 10.2 (3.2); MD -3.2, *P* < 0.001; ICIQ-LUTSqol, I: 28.8 (6.4); C 34.1 (6.7); MD -4.6, *P* = 0.005; PGI-I, *P* < 0.001; UI episode frequency (*P* = 0.001); Use of incontinence aids (*P* = 0.023).  Within groups: improvement in all outcome measures for both groups, except ICIQ-LUTSqol and use of incontinence aids (C). Significant improvement for ICIQ-UI-SF in the C (MD -0.9), and significant reduction in use of incontinence aids for the I (*P* < 0.001).  At 2 years: Only I group analysed. Significant improvements in UI severity, condition-specific QoL, and decrease in incontinence aid use (ICIQ-UI-SF: MD 3.1, *P* < 0.001; ICIQ-LUTSqol: MD 4.0, *P* < 0.001; aid usage: *P* = 0.04) compared to baseline. |
| 30 | Nyström et al [73] | Mobile app – Tät | ICIQ-UI-SF  PGI-I | At 3 months: 68% reported improvement in UI symptoms. |
| 35  35a | Rygh et al [63]  Nyström et al [107] | Mobile app – Tät | ICIQ-UI-SF  PGI-I  ICIQ-UI-SF  PGI-I | At 3 months: Improvement in both outcome measures.  ICIQ-UI-SF: significant improvement by users, with score change from 8.11 (3.87) to 6.80 (3.97) of a possible 21 points, MD 1.31, *P* < 0.001. Larger reduction in score was evident with users with greater UI severity at baseline (severe/very severe: MD 3.23, P<0.001; moderate: MD 1.41, *P* < 0.001; slight: MD 0.24, P=0.010). Score change was similar across UI types (stress: MD 1.28, *P* < 0.001; urgency: MD 1.33, *P* < 0.001; mixed: MD 1.34, *P* < 0.001).  PGI-I: improvement reported by 65.2% of users, with 25.9% reporting symptoms as much or very much better. Proportions of improvement were similar across subgroups (severity: slight 61.1%, moderate 68.8%, > severe 62.0%; type of UI: stress 65.0%, urgency 66.2%, mixed 66.3%).  Compared to the results from RCT, ICIQ-UI-SF: MD 2.2 vs 3.9 in the RCT; PGI-I: 65% improvement vs 92% in the RCT.  At 3 months: Any improvement reported by 65.6% of users, of which 26.1% reported great improvement at 3 months (PGI-I). Any improvement was associated with age, and great improvement significantly associated with total ICIQ-UI-SF score (*P* < 0.001). |
| 37 | Samuelsson et al [50] | Mobile app – Tät | ICIQ-UI-SF | At 3 months: UI severity reduced in all groups, with greater reduction observed with greater leakage frequency. Mean ICIQ-UI-SF score reduction of 2.63 points (95% CI: 1.86-4.30) for all users who had leakage at baseline; 3.94 (95% CI: 3.01–4.86) reduction for users with leakage frequency > 1/week. Reported leakage frequency at baseline was 21% no leakage, 27% ≤ 1/week, 27% 2–3 times/week, 12% 1/day, and 13% > 1/day or always. |
| 8 | Bokne et al [41] | I: Internet-based program – Tät  C: Booklet guided PFMT program | ICIQ-UI-SF | At 3 months: Between groups: No evidence of significant difference; Within groups: Significant improvement in both groups using ICIQ-UI-SF, I: 3.4 (2.9), P<0.001; C: 2.6 (3.4), P<0.001 |
| 39 | Sjöström et al [6] | I: Internet-based program – Tät  C: Printed PFMT instructions via post | ICIQ-UI-SF  ICIQ-LUTSqol  EQ5D-VAS^j^  2-day bladder diaries  PGI-I | At 4 months: Between groups: significant difference in mean ICIQ-UI-SF for participants with severe leakage at baseline only (I: 8.1; C: 11.0; P=0.006), perceived improvement using PGI-I (I: 40.9%; C: 26.5%; P=0.01) and decrease in use of UI aids (I: 59.5%; C: 41.4%; P=0.02). No evidence of significant difference in other outcome measures.  Within groups: significant improvement for all outcome measures except health-specific QoL (EQ5D-VAS) in C. ICIQ-UI-SF, I: 10.4 (3.1) to 6.9 (3.1), MD 3.4 (3.4), P<0.001; C: 10.3 (3.5) to 7.3 (3.9), MD 2.9 (3.1), *P* < 0.001; ICIQ-LUTSqol, I:33.6 (6.8) to 27.8 (6.0), MD 4.8 (6.1), *P* < 0.001; C: 33.6 (8.2) to 28.8 (7.3), MD 4.6 (6.7), *P* < 0.001; UI episode frequency, I: 12.7 (12.0) to 4.8 (7.7), MD 7.6 (9.1), *P* < 0.001; C: 9.4 (8.6) to 4.4 (6.7), MD 4.5 (7.1), *P* < 0.001; EQ5D-VAS, I: 79.1 (13.6) to 83.3 (10.3), MD 3.7 (10.9), *P* = 0.001.  At 1 year: Between groups: no evidence of significant difference.  Within groups: significant improvement for both groups in ICIQ-UI-SF (I1: 10.4 [3.1) to 6.6 [3.1], MD 3.7 [3.3], *P* < 0.001; I2: 10.3 [3.5] to 6.7 [3.2], MD 3.2 [3.4], *P* < 0.001), ICIQ-LUTSqol (I1: 33.6 [6.8] to 27.5 [6.1], MD 5.5 [6.5], *P* < 0.001; I2: 33.6 [8.2] to 27.8 [5.7], MD 4.7 [6.5], *P* < 0.001), with significant decrease in use of UI aids (I1: 75% to 47.6%, P<0.001; I2: 73.4% to 57.0%, *P* = 0.001), but no significant improvement in health-specific QoL (EQ5D-VAS).  At 2 years: Between groups: no evidence of significant difference.  Within groups: significant improvement for both groups in ICIQ-UI-SF (I1: 10.4 [3.1) to 6.5 [3.0], MD 3.6 [3.5], *P* < 0.001; I2: 10.3 [3.5] to 6.4 [3.5], MD 3.4 [3.3], *P* < 0.001), ICIQ-LUTSqol (I1: 33.6 [6.8] to 26.5 [5.2], MD 6.4 [6.0], *P* < 0.001; I2: 33.6 [8.2] to 27.2 [6.4], MD 4.8 [7.6], *P* < 0.001), with significant decrease in use of UI aids (I1: 74% to 45.2%, P<0.001; I2: 72.2% to 53.2%, P<0.001), and significant improvement in health-specific QoL (EQ5D-VAS) for I1 only (from 79.1 [13.6] to 83.3 [13.2], MD 3.8 [11.4], *P* = 0.005). |
| 42 | Wadensten et al  [64] | I: Mobile app – Tät II  C: Information app | Cure-rate (no recorded leakages) ICIQ-UI-SF ICIQ-LUTSqol ICIQ-OAB^k^  Incontinence Scale  2-day bladder diary  PGI-I | At 15 weeks: Between groups: significant difference in all outcome measures.  Cure-rate, I: 32%; C: 6%; *P* = 0.002; ICIQ-UI-SF, I: 7.0 (3.7); C: 9.8 (3.5); MD: -3.1; *P* = 0.001; ICIQ-OAB, I: 4.7 (2.0); C: 6.4 (2.0); MD: -1.8; *P* < 0.001; ICIQ-LUTSqol, I: 29.8 (7.8); C: 36.5 (9.0); MD: -6.3; *P* = 0.004; Incontinence Scale, I: 2.3 (2.1); C: 4.1 (2.5); MD: -1.6; P = 0.016  2-day bladder diary^l^: reduction in UI episode frequency (I: 3.5 [0.0-10.5]; C: 10.5[7.0-21.0]; P<0.001) and UI aid usage (P = 0.01); PGI-I, improvement I: 87%; C: 30%; P < 0.01.  Within groups: significant improvement in all outcomes for both groups at 15 weeks, except ICIQ-OAB and UI aid usage for C.  ICIQ-UI-SF^m^, I: MD -4.7 (-5.7 to -3.7), P<0.001; C: MD -1.6 (-2.3 to -1.0), *P* < 0.001; ICIQ-OAB^e^, I: MD -2.1 (-2.5 to -1.6), P < 0.001; ICIQ-LUTSqol^m^, I: MD -7.7 (-9.2 to -6.2), *P* < 0.001; C: MD -1.5 (-2.9 to -0.2), P=0.031; Incontinence Scale^m^, I: MD -2.2 (-2.9 to -1.5), *P* < 0.001; C: MD -0.6 (-1.2 to 0.0), *P* = 0.037; 2-day bladder diary^l^, I: 17.5 (10.5-27.1) to 3.5 (0.0-10.5), MD -10.5 (-17.5 to -3.5), *P* < 0.001; C: 21.0 (7.0-31.5) to 10.5 (7.0-21.0), MD -3.5 (14.0 to 3.5), *P* = 0.003. UI aid usage I: *P* < 0.001. |
| 7 | Barbato et al [40] | Internet-based PFMT program | PGI-I | At 3 weeks: 85% of participants reported improvement in UI symptoms. Mean score = 2.91 (0.62); not significantly different from 3.0 (which indicates symptoms were a little better). |
| 10 | Carrión Pérez et al (2015) [43] | I: Telerehabilitation device  C: Usual care | ICIQ-UI-SF KHQ^n^  Bladder diary | At 3 months: Between and within groups: No evidence of significant difference in all outcome measures. |
| 11 | Coggins et al (2017) [44] | Mobile app, vaginal device – Elvie | ICIQ-UI-SF GPI^o^ | 62.8% of participants reported an improvement in UI symptoms after non-specified duration of app and device use. Significant relationships identified between the odds of improvement and the duration (odds ratio 2.24, *P* = 0.001) and frequency (odds ratio 2.17, *P* = 0.002) of use. |
| 12 | Conlan et al [45] | Telehealth | ICIQ-UI-SF; ICIQ-LUTSqol; PGI-I; 3-day bladder diary | At 6 weeks: All participants rated improvement in UI symptoms, with a mean ICIQ-UI-SF score of 6.8 (4.3) compared to 11 (3.8), MD 4.2 (2.6). QoL improved and UI frequency decreased for 4 participants. ICIQ-UI-LUTSqol: 35.2 (8.4) compared to 38.0 (6.0), MD 2.8 (4.4); median improvement in UI frequency of 1 episode (IQR 2.5) per 3-day period. |
| 14 | Dufour et al [67] | I: Mobile app, vaginal device – iBall  C: PFMT instructions | UDI-6^p^; IIQ-7^q^ | At 16 weeks: Between groups: No evidence of significant difference in all outcome measures. Within groups: significant improvement for urogenital distress (UDI-6) only in both groups, I: 7.3 (5.9) compared to 18.9 (11.5), *P* = 0.009; C: 4.6 (6.0) compared to 25.4 (15.9), *P* = 0.004. |
| 18 | Fischer Blosfield et al [57] | I1: iPelvis app + physiotherapy  I2: Home exercise sheet + physiotherapy  I3: iPelvis app  C: Home exercise sheet | ICIQ-UI-SF  KHQ | At 3 months: Between groups: significant difference between I1 and C in ICIQ-UI-S (*P* = 0.01. Significant difference in KHQ and overall score for general health perception (improvement in I2 score compared to C, *P* = 0.008), social limitations (improvement in I2 compared to I3, *P* = 0.04, and C compared to I3, *P* = 0.05) and overall part score 1 (improvement in I2 compared to C, *P* = 0.04).  Within groups: significant reduction in UI severity using ICIQ-UI-SF except for C, I1: MD -4.42 (4.4), *P* = 0.0009; I2: 5.5 (4.0), *P* = 0.00006; I3: -3.6 (4.0), *P* = 0.001. |
| 19 | Goode et al [58] | Web-based –MyHealth*e*Bladder | ICIQ-UI-SF  GPI | At 8 weeks: Improvement in UI symptoms exceeding the minimal clinically important difference of 2.5 points using the ICIQ-SF: 8.7 (4.0) compared to 12.6 (3.9), MD 3.9, *P* = 0.002. 25% of participants reported their improvement as “much better”, 40% as “better”, and 25% as “about the same”. |
| 22 | Hui et al [59] | I: Telemedicine continence program  C: Usual care | Patient-rated severity score; 3-day voiding diary: number of UI^r^ episodes,  voiding frequency, and volume | At 8 weeks: Between groups: no evidence of a significant difference in all outcome measures.  Within groups: significant improvement in all outcome measures for both groups.  Severity score, *P* < 0.0001 for I and C  Number of UI episodes, I: 1.3 (1.4) to 0.2 (0.2), P<0.001; C: 1.1 (1.1) to 0.1 (0.5), *P* < 0.001  Voiding frequency, I: 11 (3.3) to 8.5 (1.5), P=0.002; C: 10.7 (3.0) to 9.0 (3.7), *P* < 0.001  Voiding volume (mL), I: 155 (59) to 198 (47), P=0.008; C: 119 (35) to 159 (25), *P* < 0.001 |
| 24 | Kinouchi & Ohashi [68] | I: Smartphone-based reminder system  C: PFMT via leaflet and verbal instructions | Questionnaire | At 8 weeks: Significant difference between groups in UI prevalence, I: 0 (0.0); C: 7 (24.1)^s^; *P* = 0.004 |
| 27 | Loohuis et al [61] | I: Mobile app –URinControl  C: Usual care  1-year follow up | ICIQ-UI-SF  ICIQ-LUTSqol  Frequency volume chart  PGI-I  ICIQ-UI-SF  ICIQ-LUTSqol | At 4 months: Between groups: no evidence of a significant difference in all outcome measures at 4 months.  Within groups: improvement^t^ in all outcome measures for both groups.  ICIQ-UI-SF, I: MD -2.16(2.56); C: MD -2.56 (3.51); ICIQ-LUTSqol, I: MD -4.34 (5.44); C: MD -3.78 (5.90); UI episodes per day, I: MD -0.61 (2.02); C: -0.48 (1.2); PGI-I, I: 65.7%; C: 66.6% reported improvement  At 1 year: Between groups: No evidence of significant difference in outcome measures.  Within groups: improvement^t^ in both outcome measures for both groups.  ICIQ-UI-SF, I: MD -2.17 (2.8); C: MD -3.43 (3.6); ICIQ-LUTSqol, I: MD -4.66 (5.1); C: MD -4.34 (5.7) |
| 32 | Pla et al [49] | Mobile app, vaginal device – Birdi | ICIQ-SF  ISI^u^ | Significant difference in ISI at 2 months from baseline (*P* = 0.042), No evidence of significance in all other UI measures at 1 and 2 months. |
| 33 | Pulliam et al [62] | Mobile app, vaginal insert – Leva Pelvic Digital Health System | UDI-6; Revised Urinary Incontinence Scale  PGI-S  IIQ-7  Voiding diary | At 6 weeks: Significant improvement in all outcome measures (results of voiding diaries not provided), with benefits (*P* < 0.05) by 1 week from training onset.  UDI-6: score decrease by 96%, from 27.5±16.9 to 1.1±2.9 of a maximum 100 points, *P* < 0.0001.  Revised Urinary Incontinence Scale: decrease by 80% from 9.9±2.5 to 1.9±1.9 of a maximum 16 points, *P* < 0.0001.  PGI-S: score decrease by 87% from 1.5±0.6 to 0.2±0.5 of a maximum 3 points, *P* < 0.0001.  IIQ-7: score decrease by 99% from 17.6±21.6 to 0.2±1.0 of a maximum 100 points, *P* = 0.0009. |
| 38 | Shelly et al [76] | Mobile app, vaginal probe – PeriCoach | PFDI^v^  Voiding frequency | At 8 weeks: 40.1% improvement in PFDI score from 41.6 to 24.9 out of a possible 300 points. Voiding frequency decreased from once every 1.5 hours to once every 2.5-3.5 hours. |
| 40 | Smith [53] | I: Mobile app, vaginal probe – PeriCoach  C: Standard PFMT | IQOL^w^  24-hour pad weight test | At 20 weeks: Between groups: significant difference in IQOL subscale of psychosocial impact favouring I (*P* = 0.0165), no significant difference for other UI outcomes between groups.  Within groups: no statistically significant improvement in both outcome measures. |
| 43 | Wang et al [51] | I: Mobile app, audio guidance – Pen Yi Kang  C: Conventional home-based PFMT | ICIQ-UI-SF | At 6 months: Between groups: No significant difference between groups.  At 6 weeks, and 3 and 6 months: Within groups: Significant improvement in UI severity (ICIQ-UI-SF)^x^, I: −4.245 (0.304), −5.03 (0.208), −5.520 (0.176); C: data not specified. |

^a^Ordered alphabetically, but where relevant, are grouped by app.

^b^PFMT: pelvic floor muscle training.

^c^ICIQ-VS: International Consultation on Incontinence Questionnaire-Vaginal Symptoms.

^d^ICIQ-UI-SF: International Consultation on Incontinence Questionnaire-Urinary Incontinence-Short Form.

^e^QUID: Questionnaire for Urinary Incontinence Diagnosis.

^f^ICIQ-LUTSqol International Consultation on Incontinence Questionnaire-Lower Urinary Tract Symptom quality of life.

^g^PGI-I Patient Global Impression of Improvement.

^h^I: intervention group.

^i^C: control group.

^j^EQ5D-VAS: EuroQol 5D-Visual Analogue Scale.

^k^ICIQ-OAB: International Consultation on Incontinence Questionnaire-Overactive Bladder module.

^l^reported as median (interquartile range).

^m^reported as mean (95% confidence interval).

^n^KHQ: King’s Health Questionnaire.

^o^GPI: Global Perception of Improvement.

^p^UDI-6: Urinary Distress Inventory-6.

^q^IIQ-7: Incontinence Impact Questionnaire 7.

^r^UI: urinary incontinence.

^s^reported as number (%).

^t^significance not measured as part of within-group analyses.

^u^ISI: Incontinence Severity Index.

^v^PFDI: Pelvic Floor Distress Inventory.

^w^IQOL: Incontinence Quality of Life.

^x^data provided as regression coefficient (β) with standard error.

**Table S2. Satisfaction with, and adherence, to the PFMT programme delivered via digital technologies.**

| **ID** | **Author^a^** | **DT^b^** | **Satisfaction with programme** | | **Adherence to programme** | |
| --- | --- | --- | --- | --- | --- | --- |
|  |  |  | **Measure** | **Result** | **Measure** | **Result** |
|  | | | | | | |
| 1 | Anglès-Acedo et al [37] | Mobile app – WOMEN UP | Pleased or very pleased with the ICT^c^ system | 80% | Adherence (not defined) | Mean 70.4% (range 41.8-86.2%) for the 15/21 women who completed the programme |
| 3 | Araujo et al [39] | Mobile app – Diário Saúde | Would recommend app to others Attributed success of treatment to the app | 75% 90% | Number of protocol repetitions (one repetition = completion of the entire 8 reps of a PFMT^d^), Mean (SD)  Self-reported adherence (VAS^e^ 0-10; 10 = maximal adherence), Mean (SD) | At 1 month: I^f^ = 52.9 (5.5); C^g^ = 43.7 (11.1) At 2 months: I = 49.8 (8.1); C= 33.6 (10.7) At 3 months: I = 43.8 (8.7), Control = 17.7 (6.3) Significantly higher in I compared to C, at 1 and 2 months  At 1 month: I = 9.5 (0.7); C = 8.3 (1.5) At 2 months: I = 9.9 (0.2); C = 9.0 (1.3)  At 3 months: I = 9.9 (0.2); C = 8.7 (1.3)  Significantly higher in I compared to C at all time points |
| 4 | Asklund et al [5] | Mobile app – Tät | Satisfaction with app (5 responses from "very bad" to "very good" | Good or very good = 96.7% (59/61) | App use  Mean number of exercises over 3 months, recorded by women who used the statistics function  Self-reported PFMT, at 3 months | I: All women who completed follow-up (n = 61) had used the app  1.6 exercises/day (141 exercises/person for 51 women)  During last 4 weeks: I = 98% (60/61)  Daily: I = 41.0% (25/61) Weekly but not daily PFMT: I = 42.6% (26/61) Sporadic: I = 14.8% (9/61)  Daily: C = 3.3% (2/60) Weekly but not daily: C = 13.3% (8/60) Sporadic PFMT: C = 56.7% (34/60) No PFMT: C = 26.7% (16/60) |
| 4e | Nyström et al [79] | Mobile app – Tät | Perception of outcome after treatment (7 responses from "very much worse" to "very much better"; collapsed into two outcomes of successful or not successful) | Successful treatment, defined as much or very much better = 55.7% (34/61)  Not successful treatment, defined as the remaining responses = 44.3% (27/61) | Self-reported PFMT, at 3 months, for those who self-reported a successful outcome  Self-reported PFMT, at 3 months, for those who self-reported an unsuccessful outcome | During last 4 weeks Daily: 47.1% (16/34) Weekly but not daily PFMT: 41.2% (14/34) Sporadic or never: 11.8% (4/34)  Daily: 33.3% (9/27) Weekly but not daily PFMT: 44.4% (12/27) Sporadic or never: 22.2% (6/27) |
| 4g | Asklund 2019 [78] | Mobile app – Tät | Satisfaction with treatment effect (yes/no) | 66.7% (40/60) | – | – |
| 35 | Rygh et al [63] | Mobile app – Tät | – | – | Self-reported PFMT, frequency in past 4 weeks (all users^h^)  Self-reported app usage, in past 3 months (all users) | Never = 8.8% (244/2672) < 1 x/week = 24.3% (650/2672) 1–6 x/week = 37.7% (1008/2672) Daily = 23.4% (624/2672) ≥ 3 times/day = 5.5% (146/2672)  Never = 11.0% (295/2672)  About 1x/month = 14.3% (383/2672) About 1x/week = 22.7% (607/2672) About 1 x/day = 27.9% (745/2672) Several times a day = 24.0% (642/2672) |
| 37 | Samuelsson et al [50] | Mobile app – Tät | – | – | Self-reported PFMT, past 3 months Self-reported app use, past 3 months | ≥ weekly = 69% (188/273)  Used the app = 89% (243/273) |
| 8 | Bokne 2019 et al [41] | Internet-based program – Tät | – | – | Self-reported PFMT frequency, last 4 weeks (months 2–3 of the programme) | > 3 times/week: I = 40.7% (11/27); C = 58.5% (31/53) (p = 0.34) |
| 39 | Sjöström et al [6] | Internet-based program – Tät | Satisfaction with the treatment programme (5 options ranging from ‘very good’ to ‘very bad’; at 1- and 2-year follow-up 4 responses from "very dissatisfied' to 'very satisfied') | At 4 months (good or very good): I = 84.8% (89/105, 95% CI 76.9–90.7); C = 62.9% (71/113, 95% CI 53.7–71.4); Significantly higher in I  At 1 year (satisfied): I = 69.8% (60/86); C = 60.5% (46/76); Not significantly different  A 2 years (satisfied): I = 64.9% (48/74); C = 58.2% (46/79) Not significantly different | Training diary, PFMT frequency | – |
| 15 | Firet et al [56]^i^ | Internet-based program – Tät | – | – | Self-reported PFMT frequency  Adherence to programme (progress through modules in app) | – |
| 42 | Wadensten et al [64] | Internet-based program – Tät | Satisfaction (3 responses relating to satisfaction and intention to seek further care)  Satisfaction with app content and usability (scale not defined – ranked 1, 2 or 3) | Satisfied and symptom-free = 7% (4/60)  Satisfied, with some symptoms = 51% (31/60)  Not satisfied = 33% (20/60); of these 35% (7/20) would seek additional care  Most participants satisfied with app (contents and usability)  PFMT portion = 95% (57/60) Exercise log = 80% (48/60) Tailored advice = 66% (40/60) | App use, recorded by women who used the statistic function | > 3 x week = 67% (40/60) ≥ 3 times/day = 10% (6/60) |
| 7 | Barbato et al [40] | Internet-based program | Self-rated improvement in SUI (7 responses from "very much worse" to "very much better")  Use of the technology – agreed or strongly agreed | Much better = 23.5% (8/34) A little better = 61.8% (21/34) No change = 14.7% (5/34)  Website easy to access = >94% (32/34) Videos easy to access = 85.3% (29/34) Instructions easy to understand = 88.2% (30/34)  Would recommend programme to others = 97.1% (33/34) | – | – |
| 9 | Campbell et al [42] ^i^ | Mobile app – Squeezy App | Qualitative process evaluation (Phase 3): Acceptability of intervention; use of Squeezy app | – | Attendance (number of scheduled appointments attended; Phase 2)  PFMT frequency, app usage | – |
| 34 | Robson [74] | Mobile app – Squeezy App | User experience of various aspects of the app (Likert Scale, 0–10; 10 = highest) | Overall user experience: rated 8–10/10 = 86% (400/464) Popularity of reminder feature: ≥ 8/10 = 75% (348/464) | Self-reported PFMT, frequency/week    PFMT frequency, when received reminder | 6-7 days = 47% (218/464) 3–5 days = 33% (153/464) 1–2 days = 14% (65/464) None = 7% (28/464)  Always = 39% (180/464) Sometimes = 54% (251/464) Rarely = 5% (23/464) Never = 2% (10/464) |
| 10 | Carrión Pérez et al [43] | Telerehabili-tation device, vaginal probe | Satisfaction with treatment programme (VAS) | I: 8 (6.3–9.5) C: 8 (7.5–8.5) No difference | Adherence to PFMT (days/week), recorded through device | Intervention (n = 10): Median 4.9 (range 4.2–6.3) Control (n = 9): Median 7 (range 2.5–7) No statistically significant different between groups |
| 11 | Coggins et al [44] | Mobile app, vaginal device – Elvie | Degree of satisfaction (would recommend app to others; 0 = not at all likely to 10 = extremely likely)  Self-reported improvement in symptoms | ≥ 8 = 67.8% (283/417)  A little better or very much better = 62.8% (262/417) Very much better = 10.3% (43/417) | Self-reported frequency of PFMT | Prior to purchasing Elvie: ≥ 1 x week = 25.9% (108/417) After purchasing Elvie: ≥ 1 x week = 77.0% (321/417) Significant difference reported between the two time-points |
| 12 | Conlan et al [45] | Telehealth | Satisfaction with the telehealth | Agreed or strongly agreed = 83% (5/6) | – | – |
| 13 | Cornelius [71] | Mobile app, vaginal probe – PeriCoach |  | Patient very satisfied with treatment [89] | Compliance (completed at least 75% of programme requirements) | 14.4% (38/265) [91] |
| 14 | Dufour et al [67] | Mobile app, vaginal device – iBall | Would recommend mHealth device or consider using it again  Consider using device again with modifications | 18.2 % (2/11)  63.6% (7/11) | App usage | –^j^ |
| 19 | Goode 2020 [58] | Web-based – MyHealtheBladder | Self-reported satisfaction with treatment  Perception of improvement with treatment (5 responses from "much worse" to "much better") | Completely satisfied = 19% (4/20) Somewhat satisfied = 75% (15/20) Not satisfied = 6% (1/20)  Much better = 25% (5/20) Better = 40% (8/20) About the same = 35% (7/20) | Self-reported number of sessions performed daily | 20/29 (69%) women completed ≥ 80% of the 56 daily sessions over 8 weeks (completers)  Mean (SD) adherence to the 8-week programme was 97 (4.9)% among completers |
| 21 | Han et al [72] | Mobile app – Bwom | Understandability and actionability of the app (Patient Education Materials Assessment Tool (PEMAT)) | Understandability (Mean SD): 93.8 (11.7)%  Actionability (Mean, SD): 91.7 (16.3)% | – | – |
| 22 | Hui et al [59] | Videoconferenc-ing | Self-reported satisfaction with the telemedicine programme (6-point Likert Scale, 0 = highly dissatisfied to 5 = highly satisfied) | 5 = 100% (27/27) | – | – |
| 23 | Jaffar et al [60] ^i^ | Mobile app – KEPT-app^k^ | Acceptability and feasibility of the app (no measures provided) | – | Adherence, defined as completing 80% of the expected training time (as logged by the participants in the app) | – |
| 24 | Kinouchi & Ohashi [68] | Smartphone-based messaging system | – | – | Self-reported PFMT implementation rate (% who self-reported performing PFMT over 8 weeks)  Self-reported training intensity (number of pelvic floor muscle contractions/day), Median (IQR)  Self-reported training frequency (PFM contractions/week), Median (IQR) | I = 69% (20/29); C = 31% (9/29)  I = 15 (3-8); C = 1 (1-4)  I = 7 (6-7); C = 3 (1-4) Significant differences between groups for all measures (in favour of I) |
| 28 | Moossdorff-Steinhauser et al [52] ^i^ | Mobile app – iPelvis (in conjunction with the Motherfit programme) | Satisfaction with the Motherfit group therapy and use of the mApp (Likert Scale; 1-5) | – | Training diary to capture performance of PFM exercises (weekly) | – |
| 25 | Li et al [69] | Mobile app, audio guidance – Pen Yi Kang | – | – | Participation in PFMT (High = PFMT ≥ 15 times; low = PFMT < 6 times), app use | High = 35.2% (262/745); Low = 64.8% (483/745) |
| 43 | Wang et al [51] | Mobile app, audio guidance – Pen Yi Kang | – | – | Broome Pelvic Muscle Self-Efficacy Scale (23 items across 2 domains – efficacy expectations and outcome expectations; score 0-100; higher better) | Mean difference of 8.9 points at 6 months postpartum, favouring I |
| 26 | Li et al [47] ^i^ | Mobile app – UIW^l^ | – | – | PFMT adherence and activity, usage results and patterns; self-administered, electronic PFMT diary; and an electronic questionnaire | – |
| 27 | Loohuis et al [61] | Mobile app – URinControl |  |  | App use and PFMT | Used the app at least once = 94% (96/102) |
| 29 | Moretti [36] | Mobile app, vaginal probe, surface electrodes – MyoPelvic | Satisfaction with system (VAS), Median (IQR)  Satisfaction with the virtual game app (VAS), Median (IQR)  Usability (MATCH), Mean (SD) | 9 (8–10)  9 (9–10)  62.4 (5.0) points^m^ | – | – |
| 32 | Pla et al [49] | Mobile app, vaginal device – Birdi | Satisfaction with treatment | 100% (7/7) | – | – |
| 33 | Pulliam 2018 [62] | Mobile app, vaginal insert – Leva pelvic digital health system | User friendliness (0-10; easiest-impossible), Median (range) | 2.0 (1.4–2.6) | – |  |
| 44 | Weinstein et al [54] ^i^ | Mobile app, vaginal insert – Leva pelvic digital health system | Willingness to recommend the treatment regime (VAS scale) | – | Percentage of time exercises performed, 4 and 8 weeks (both groups) (VAS) Number of completed exercise episodes/day (I), from the device | – |
| 36 | Saboia et al [75] | Mobile app – Continence App | Content validation indexes rated by 14 health experts (5 items)  Appearance validation rated by health experts and ICT/computing experts and users | > 0.90 for all items  Most items received between 75–100% positive responses | – | – |

^a^Studies are ordered alphabetically by first author, but where relevant, are grouped by app.

^b^DT: digital technologies.

^c^ICT: information and communication technologies.

^d^PFMT: pelvic floor muscle training.

^e^VAS: visual analogue scale.

^f^I: intervention group.

^g^C: control group.

^h^also report data separately for women with and without urinary incontinence.

^i^study protocol, no reported data.

^j^analytics data combined with interview data highlighted the lack of adherence to the intervention protocol.

^k^KEPT: Kegel Exercise Pregnancy Training.

^l^UIW: Urinary Incontinence for Women.

^m^indicates high level of usability.
