# Supplementary material for: Digital Technologies for Women’s Pelvic Floor Muscle Training to Manage Urinary Incontinence Across Their Life Course: Scoping Review
Source: JMIR Mhealth Uhealth. 2023 Jul 5;11:e44929. doi: 10.2196/44929 (PMC10357376; doi:10.2196/44929)
Supplement: Multimedia Appendix 5 [file mhealth_v11i1e44929_app5.docx]

**Multimedia Appendix 5.**

**Table S1. Summary of main themes, and facilitators and barriers from qualitative studies (*N*=11).**

| Outcomes | | | | | |
| --- | --- | --- | --- | --- | --- |
| Article | | **Study** | | | |
| ID | **Author^a^** | **DT^b^ type,**  **Group(s)** | **Main themes** | **Facilitators** | **Barriers** |
|  | | | | | |
| 2 | Anglès-Acedo et al [38] | Mobile app – WOMEN UP  Stage 1 (n = 22; women with UI^c^): Open-ended questions from questionnaire  Stage 3 Usability testing (n = 9; two with UI) -Phase 1: Women tested app usability while thinking aloud with a HCP^d^ -Phase 2: Women used app at home for 2 weeks  -Phase 3: think-aloud testing and retrospective interview to explore usability, functionality, satisfaction, and preference | -Global functionality^e^ -Mechanical design -System features -Device instructions -Feedback on exercises -Patient-professional interaction preferences -System interface -Serious games  Stage 3 – Facilitators and barriers presented with ideas to be incorporated into ongoing design upgrades prior to use of WOMEN-UP in future intervention studies | *Interactions between users and DT*  -Provided autonomy and independence  -Positive feedback about portability of app  -Calibration of device was easy-to-learn  -Device was comfortable  *Interactions between users and PFMT^f^*  -Not presented  *Interactions between PFMT and DT*  -Choice of games to provide PFMT simulated biofeedback  -Good control during games (relating to PFMT)  -Games made PFMT programme fun | *Interactions between users and DT*  -Not all functionalities were in app  -Outdated design  -Use of medical jargon (e.g., PFMT)  -Not all app navigation buttons could be used  -Design of some buttons could be improved  -Poor visualisation of results on the web platform  -Discomfort with some devices  -Lack of available instructions  -Difficulties with web accessibility of instructions  -Issues with device connectivity with limited assistance available to resolve issues  -Difficulties with calibration with limited means of getting assistance  -Some games and menus unclear  -Limited encouragement and increasing difficulty levels in some games  -Issues with receiving messages from the therapist or lack of awareness that these had occurred  *Interactions between users and PFMT*  -Not presented  *Interactions between PFMT and DT*  -No or difficult to interpret feedback to evaluate performance of PFM^g^, performance of PFMT or progress of PFM contraction  -Gaming biofeedback difficult due to score visualisation  -Training schedule names were confusing  -Naming sessions as “incomplete” was de-motivating |
| 4g | Asklund et al [78] | I^h^: Tät app (n = 15)  C^i^: No intervention (n = 0)  Individual telephone interviews 3-4 months after completion of last RCT questionnaire | Core category: Enabling my independence  Three sub-categories: 1. Something new! 2. Keeping motivation up! 3. Good enough? | *Interactions between users and DT*  -App convenient and easy to use  -PFMT program was fixed but progression could be individualised  -Visual screen and audio reminders to exercise were helpful  -Women preferred to use app, even if they knew the PFMT, as it helped them stay focused on the exercise  -Decreased the need for in-person HCP visits  *Interactions between users and PFMT*  -Empowerment: able to manage their UI independently  -App seen as “first-line” treatment, to try first, before seeking other help, if necessary  -Worth “giving it a chance” -Increased confidence to perform PFMT  -Improvement in continence symptoms after 3 months was sufficient feedback to know intervention had been performed ‘correctly’  -Satisfied at personal attempt to self-manage and felt enabled to advocate for future HCP support  *Interactions between PFMT and DT*  -Interactive graph providing real time feedback helped women see intensity and duration of contractions | *Interactions between users and DT*  -Women unsure if outcomes were equal to other intervention options  -Limited feedback increased uncertainty on individual ability to perform PFMT programme ‘correctly’  *Interactions between users and PFMT*  -Prioritisation of exercise programme over competing life events  -Hard to maintain enthusiasm over time especially as limited variation in PFMT content over time  -Improved UI symptoms decreased motivation  -Preference to seek HCP support after no change in outcome at 3 months  *Interactions between PFMT and DT*  -Even with reminders initiating PFMT could be challenging due to competing priorities |
| 39b | Björk et al [86] | I1: Internet-based program (Tät), with email urotherapist (n = 13)  I2: Printed PFMT instructions via post (n = 8)  One telephone interview | Four main themes  1. Hidden but present (aspects of life with incontinence and the factors that led to participation in the RCT) 2. At a distance but close (experiences of the treatment programs and the patient-provider relationship) 3. By myself but not alone 4. Acknowledged but not exposed (Combining all 3 categories) | *Interactions between users and DT*  -Internet-based programme makes it easier to seek care  -Anonymity offered by programme increased sense of control as women were able to determine pace and the focus  -Positive ‘non-judgemental’ relationship with urotherapists established. Women felt acknowledged and supported without being exposed  *Interactions between users and PFMT*  **-**Both online and postal groups had increased awareness of PFMT  -Users felt empowered by content and engaging in PFMT  -Positive change in UI symptoms or knowledge in both groups  -Increased motivation to do PFMT with increasing severity of UI  -Post-intervention both groups thought about UI more constructively  -Connecting PFMT to pre-existing activities improved long term adherence as PFMT became part of the daily routine  *Interactions between PFMT and DT*  -Consistent contact with the same urotherapist increased motivation to do PFMT  -CBT component on lifestyle change, minimization of avoidance and redundant leakage related security measures was helpful  -Follow-up by HCP if PFMT report was not submitted by user each week was motivating | *Interactions between users and DT*  -Relationship with HCP was harder to establish and felt superficial  -Harder to discuss complex issues  *Interactions between users and PFMT*  -Would prefer an initial in-person consultation to reassure that everything looked normal, and to confirm that PFMT was being performed ‘correctly’  -As leakage improved motivation decreased  *Interactions between PFMT and DT*  -Sense of guilt because of the mismatch between individual goals for PFMT & actual achievements  -Follow up by HCP if PFMT report not submitted by user was stressful |
| 15a | Firet et al [31] | ‘Baas over je blaas’ (‘Master your own bladder’) – web-based, Dutch translated Tät app adaptation  Individual interview with 20 Health users | EHealth is appropriate for some users | *Interactions between users and DT*  -Accessible, anonymous, free of charge and pace of delivery determined by individual  -Easy to navigate with good layout  -Reminders were supportive  -Did not have to seek HCP consultation first  -Trusted as from credible source  -Combination of DT with personal contact: remote contact (telephone, chat or videocall) sufficient for most users  *Interactions between users and PFMT*  -Exercises were easy to perform  -Pre-existing knowledge was beneficial  -Increased severity or burden of UI motivated ongoing PFMT  -‘Self-disciplined’ users had increased adherence to PFMT  *Interactions between PFMT and DT*  -Clarity and reassurance: content (audio, image, text, test exercise)  -Task performance: functionalities (e.g., training report)  -Improvement in UI symptoms increased motivation to use DT and continue PFMT programme  -Stepwise progression of PFMT as UI symptoms improved | *Interactions between users and DT*  - Limited usability: high information density and login problems -Limited ability to ask questions or receive feedback on PFM performance  -Diagnostic self-assessment (2 women consequently consulted a HCP)  -No personal contact with HCP provided. Users felt external support and encouragement missing.  -Email reminders were overwhelming and created a sense of guilt (if users had not been doing their PFMT) -Mobile app preferred over a website  *Interactions between users and PFMT*  -Self-efficacy required to independently integrate into daily routine  -Competing priorities. PFMT not always completed due to individual circumstances or life events  -PFMT expectations i.e. frequency and the training position (e.g., lying) not always possible -‘Lack of self-discipline’ prevents self-management of PFMT  -No HCP feedback to ensure PFMT being performed ‘correctly’  -Decreased UI burden decreased motivation for PFMT  *Interactions between PFMT and DT*  -3 months access was too short with women not finishing their PFMT programme  -3 months access was too long with users losing motivation  -Limited change in UI symptoms decreased motivation to continue PFMT and trial  -Not all DT functions found or tried by all users (e.g., training report, ability to change duration of PFMT sessions) |
| 14 | Dufour et al [67] | iBall mHealth device  I: iBall (n = 13)  C: PFMT instructions (n = 10)  Acceptability and feasibility explored with individual interviews | Three main themes  1. iBall is an acceptable concept to support PFMT 2. Limited acceptability of iBall in current form 3. Numerous technology issues with iBall; newer versions may overcome these | *Interactions between users and DT*  -Biofeedback was helpful (4/13; 31%)  *Interactions between users and PFMT*  -Tracking progress was helpful (2/13; 15%)  -Instructions easy to follow and the purpose, clear (3/13; 23%)  -The device was comfortable (5/13; 38%)  *Interactions between PFMT and DT*  -Concept of iBall to help PFM rehabilitation through feedback is good (10/13; 73%)  -iBall and app provided motivation to do PFMT (4/13; 31%)  -iBall and app were helpful when combined with a PF examination (4/13; 31%) | *Interactions between users and DT*  -The device was uncomfortable (8/13; 62%)  -Biofeedback was not helpful (8/13; 62%)  -Tracking progress was not helpful (7/13; 54%) -Instructions not straight forward and the purpose, unclear (7/13; 54%) -Technical difficulties with app (6/13; 46%) -Setup of device and app was cumbersome (not new mum friendly) (8/13; 62%) -Optimal positioning of the device was an issue (5/13; 38%)  *Interactions between users and PFMT*  -Nil presented  *Interactions between PFMT and DT*  -The device and app made it more difficult to do pelvic floor exercises (barrier) (8/13; 62%)  -Initial instruction from the practitioner was more helpful than feedback from iBall device and app (9/13; 69%) |
| 16 | Firet et al [80] | Individual interviews with 13 GPs about perceptions of eHealth | Three main themes: 1 Appraisal of eHealth as a welcome new tool 2 Mixed feelings about support provided by eHealth 3. eHealth is no cure-all | *GP perspective*  *Interactions between users and DT*  -Flexibility of care delivery and ease of use may improve access to care for women who would otherwise not seek help -Financially attractive -May save consultation time in their clinical practice  -Provides an evidence based, non-pharmaceutical alternative  *Interactions between users and PFMT*  -Nil presented  *Interactions between PFMT and DT*  -Enables supported self-management  -Physiotherapists should be used in conjunction with EHealth to ensure appropriate assessment, diagnosis, and then only if appropriate, individualised PFMT is initiated  -Ongoing follow-up by HCP should be provided | *GP perspective*  *Interactions between users and DT*  -Initial consultation to ensure PFMT is appropriate is required  -Older users would find eHealth technology a barrier  -Aware of individual personal factors that might influence accessibility and acceptability of EHealth  -Presence of comorbidities and or complex presentations mean eHealth can be unacceptable  *Interactions between users and PFMT*  -Inability to check PFMT being done ‘correctly’  -Might discourage users from seeking face-to-face help  -Preference to use physiotherapists over EHealth  *Interactions between PFMT and DT*  -May limits individualised support for PFMT |
| 17 | Firet et al [46] | 13 face-to-face interviews with women with SUI about perceptions of eHealth | Two main themes  1. Need to meet 2. eHealth as a tool to bridge obstacles | *Interactions between users and DT*  -Improves access to PFMT  -Decreased shame and stigma as anonymous  -Flexible: used when and where individuals deem it appropriate and within their personal schedule  -Increased stress UI knowledge  -Viable option if GP trivialises PFMT  -Use of DT should occur via shared decision making  *Interactions between users and PFMT*  -Ability to do PFMT in own time  -Lack of embarrassment as decreased need to visit GP (again)  *Interactions between PFMT and DT*  -Reminders and tips on how to integrate PFMT into daily life  -Motivational interviewing with GP prior to using DT may improve daily use of app and performance of PFMT | *Interactions between users and DT*  -Technology challenges  -No personal contact with a HCP  -Users preferred face-to-face as previously had good experience with a HCP  -Needed a HCP point of contact if problems arose  -Distrust of digital therapies  *Interactions between users and PFMT*  -No HCP feedback to ensure PFMT being performed ‘correctly’  *Interactions between PFMT and DT*  **-**Decreased motivation with no external HCP feedback  -Limited trust that the technology will work |
| 20 | Grant & Currie [77] | Not technology    Focus group interviews on postnatal physical activity and PFMT intervention with 31 participants  In three focus groups there was ≥ 1 woman who had used the Squeezy app | Three main themes 1. Perceptions and experiences of engagement in postnatal physical activity 2. Perceptions and experiences of postnatal pelvic floor muscle training.  -All but one woman was aware they should be doing PFMT after having a baby 3. Potential postnatal intervention features | *Interactions between users and DT*  -An app is a good idea  -NHS approved and branded app recognised as a credible source  -Smartphone-based app preferred as always close by  -Features should include benefits of maintaining PFMT, diagrams of muscles involved and a tracking system to monitor progress  *Interactions between users and PFMT*  -Benefits of PFMT should be discussed more widely in society minimising the perception of it being a ‘taboo’ topic  -PFMT should be taught to women before they have children    *Interactions between PFMT and DT*  -An app would be useful to remind users to do PFMT programme | *Interactions between users and DT*  -Presumed app would teach how to perform a ‘correct’ PFM contraction  *Interactions between users and PFMT*  -Leaflets provided at previous HCP consultations but no women had ever been taught a ‘correct’ contraction  *Interactions between PFMT and DT*  -Women wished to be taught a ‘correct’ PFMT first |
| 31 | Pedofsky et al [48] | femfit® intravaginal device  Focus groups, individual interviews with 10 users | Four main themes  1. Engagement  2. Functionality  3. Aesthetics  4. Information | Key criteria of femfit® defined as: intuitive, reliable, usable, motivational, with good aesthetic. Women wanted the app to feel familiar, and convey a supportive environment  *Interactions between users and DT*  -Engagement: App content engaging  -Functionality: App easy to learn how to use; Practice pages enable women to understand how to do PFMT; App is easily navigable; Content easy to find  -Aesthetics: App visually appealing and fun  -Information: App content is correct, easy to understand, relevant, comprehensive yet concise  *Interactions between users and PFMT*  -Nil presented  *Interactions between PFMT and DT*  -No in-person meetings respected competing priorities of women’s lives  -Possibility of an online femfit® community for peer support. However, consideration of anonymity, sharing of information, and management of forum behaviour needed | *Interactions between users and DT*  -Biofeedback data too complicated  **-**Limited links to additional resources on the femfit® website especially instructions (insertion, practice, exercises) and app user guides  -Limited customisation for individual preferences (e.g., sound, content, notifications)  *Interactions between users and PFMT*  -Limited peer support  *Interactions between PFMT and DT*  -No online portal to enable HCP (PF physiotherapists or personal trainers) to monitor individual PFM pressure data and customise exercise programs |
| 45 | Wessels et al [65] | URinControl app    Semi-structured interviews with 9 women using app for UI treatment | Four main themes 1. Accessibility 2. Awareness 3. Usability 4. Adherence | *Interactions between users and DT*  -App may lower barriers to seeking treatment  -Privacy maintained as anonymous  -No disclosure to HCP or need for internal examination required  -App less burdensome than ongoing HCP treatment  -Flexible: used when and where individuals deemed it appropriate and within their personal schedule  -Useful for reducing UI symptoms  -Increased self-awareness of UI symptoms and potential coping and therapeutic strategies  -Changed beliefs about potential causes of UI  -Diverse group of women reported that the app was easy to use and self-explanatory  -Automated reminders helpful for motivation  *Interactions between users and PFMT*  -Not presented  *Interactions between PFMT and DT*  -Good information about the PFMT exercises  -PFMT information conveniently available so users could perform PFMT when suitable  -Videos decreased isolation when performing PFMT | *Interactions between users and DT*  -More features (games) to maintain engagement  -Increased self-awareness of severity and impact of UI  -Increased awareness of ‘dysfunctional’ coping strategies for UI symptoms  *Interactions between users and PFMT*  **-**No HCP feedback to ensure PFMT being performed ‘correctly’  -Support and guidance from a physiotherapist would add value to the app  -Decreased adherence to PFMT programme in the absence of HCP face-to-face contact  -PFMT expectations (frequency) were difficult to meet  -Competing priorities. PFMT not always completed due to individual circumstances or life events  -Decreased UI severity/burden decreased motivation for PFMT  *Interactions between PFMT exercises and DT*  -Limited feedback on improvement of PFM contractions  -Limited features to support ongoing PFMT programme  -Limited features to progress PFMT in women who had used PFMT exercises previously or had more control  -Needed privacy and space to perform PFMT programme |
| 27f | Wessels et al [87] | URinControl app    Individual interviews with users who experienced treatment success (n = 9) and treatment failure (n = 8) with app | Four main themes, with subthemes also noted here 1. Adherence: integration of exercises, level of symptoms, time investment 2. Personal factors: app vs caregiver, personality traits 3. App factors: intensive treatment, ease of use, app features 4. Awareness: education, awareness of symptoms  Cross-thematic analysis  -Adherence was a barrier and facilitator and influenced app-based treatment effect  -Adherence was influenced by personal factors, app factors, and self-awareness themes  -Awareness was facilitated by the treatment effect and by app factors. | *Interactions between users and DT*  -Liked the concept of 24-hour care independent of HCP  -App decreased need to disclose UI symptoms  -App easy to use with clear instructions  -Intensive and extensive PFMT programme went beyond care any individual HCP might usually provide  -Educational content helpful  -Decreased feelings of isolation from prevalence of UI information  -Decreased sense of a ‘taboo’ topic  -Changed understanding and increased confidence to manage UI  -Increased knowledge about alternative therapies and how to manage UI symptoms  -Increased self-awareness of UI symptoms and potential coping and therapeutic strategies  -Increased confidence to make lifestyle choices  *Interactions between users and PFMT*  -Self-management requires a ‘go-getter’ attitude  -Increased motivation to do PFMT with increasing severity of UI symptoms  *Interactions between PFMT and DT*  -Reminders supported completion of PFMT at set times enabling the establishment of a routine that worked within their schedule  -Features (e.g., graphs) provided insight about progress  -Increased self-awareness of severity and impact of UI supported motivation for PFMT programme  -Increased self-awareness  -Increased knowledge about therapy enhanced motivation for self-management  -Symptom recurrence encouraged we use of the app for PFMT  -Success with app compared to previous HCP consultations increased motivation to continue with app | *Interactions between users and DT*  **-**App interface was complex  -Difficult to identify where to start  -Difficult to get an overview of the content  *Interactions between users and PFMT*  -Requires a ‘go-getter’ attitude  **-**Would like feedback to ensure performing PFMT ‘correctly’  -Support and guidance from a physiotherapist would add value to the app  -Decreased adherence to PFMT in the absence of HCP face-to-face contact  -PFMT programme expectations (frequency and duration) were difficult to meet  -Competing priorities. PFMT not always completed due to individual circumstances or life events  -Decreased severity or burden of UI decreased motivation for PFMT programme  *Interactions between PFMT and DT*  -Reminders for completion of PFMT exercises at set times were inconvenient and at times intrusive  -Reminder features not found by all users  -Features (e.g., graphs) providing insight about progress were not found or applied by all users  -Graphs were difficult to understand or too confrontational  -‘Failure’ with app generated negative self-talk  -Increased self-awareness of UI severity and internal focus decreased motivation to use app for PFMT |

^a^Studies are ordered alphabetically by first author, but where relevant, are grouped by app.

^b^DT: digital technologies.

^c^UI: urinary incontinence.

^d^HCP: healthcare professional.

^e^Each of these topics also included recommendations and risks.

^f^PFMT: pelvic floor muscle training.

^g^PFM: pelvic floor muscles.

^h^I: intervention group.

^i^C:control group.

This table only reports the outcomes of completed studies, so it does not include two study protocols for planned/ongoing qualitative research [42, 47].
